# Sulfur-Doped g-C_3_N_4_ Heterojunctions for Efficient Visible Light Degradation of Methylene Blue

**DOI:** 10.1021/acsomega.3c06320

**Published:** 2023-12-05

**Authors:** Andrés F. Pérez-Torres, Diego F. Hernández-Barreto, Valentina Bernal, Liliana Giraldo, Juan Carlos Moreno-Piraján, Edjan Alves da Silva, Maria do Carmo Martins Alves, Jonder Morais, Yenny Hernandez, María T. Cortés, Mario A. Macías

**Affiliations:** † Crystallography and Chemistry of Materials, CrisQuimMat, Department of Chemistry, Universidad de los Andes, Bogotá D.C. 111711, Colombia; ‡ Facultad de Ciencias, Departamento de Química, Grupo de Investigación en Sólidos Porosos y Calorimetría, Universidad de los Andes, Bogotá D.C. 111711, Colombia; § Facultad de Ciencias, Departamento de Química, Grupo de Calorimetría, Universidad Nacional de Colombia, Sede Bogotá 01, Bogotá D.C. 111321, Colombia; ∥ Electron Spectroscopy Lab (LEe-), Instituto de Física, Universidade Federal do Rio Grande do Sul (UFRGS), Avenida Bento Gonçalves, 9500, 91501-970 Porto Alegre, RS, Brazil; ⊥ Instituto de Química, Universidade Federal do Rio Grande do Sul (UFRGS), Avenida Bento Gonçalves, 9500, 91501-970 Porto Alegre, RS, Brazil; # Department of Physics, Universidad de los Andes, Bogotá D.C. 111711, Colombia; ∇ Departamento de Química, Universidad de los Andes, Bogotá D.C. 111711, Colombia

## Abstract

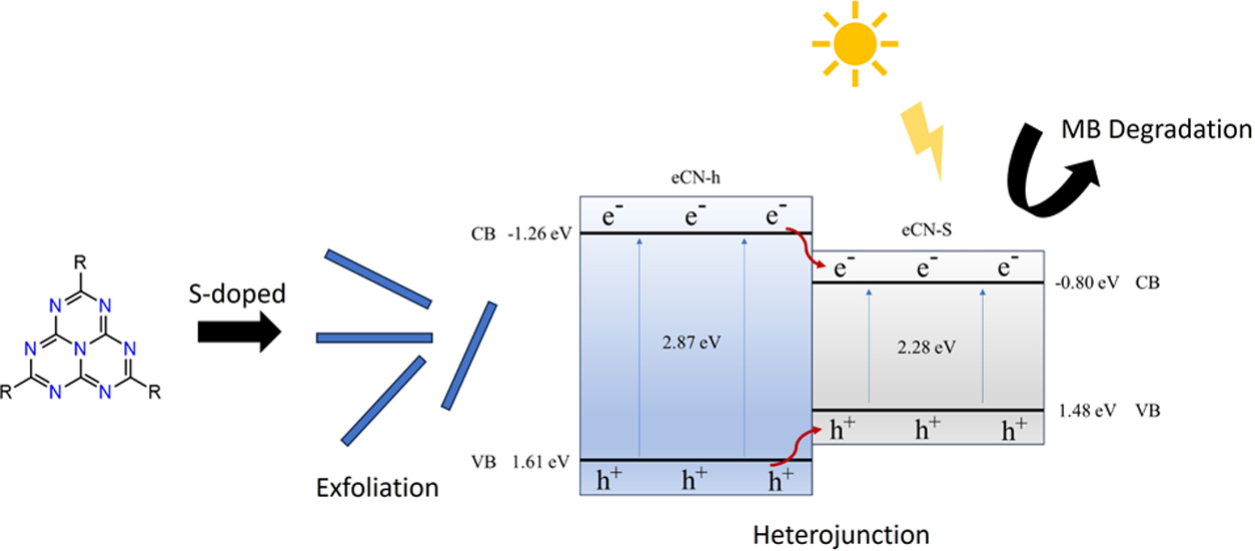

The discharge of synthetic dyes from different industrial sources has become a global issue of concern. Enormous amounts are released into wastewater each year, causing concerns due to the high toxic consequences. Photocatalytic semiconductors appear as a green and sustainable form of remediation. Among them, graphitic carbon nitride (g-C_3_N_4_) has been widely studied due to its low cost and ease of fabrication. In this work, the synthesis, characterization, and photocatalytic study over methylene blue of undoped, B/S-doped, and exfoliated heterojunctions of g-C_3_N_4_ are presented. The evaluation of the photocatalytic performance showed that exfoliated undoped/S-doped heterojunctions with 25, 50, and 75 mass % of S-doped (g-C_3_N_4_) present enhanced activity with an apparent reaction rate constant (*k*_app_) of 1.92 × 10^–2^ min^–1^ for the 75% sample. These results are supported by photoluminescence (PL) experiments showing that this heterojunction presents the less probable electron–hole recombination. UV–vis diffuse reflectance and valence band-X-ray photoelectron spectroscopy (VB-XPS) allowed the calculation of the band-gap and the valence band positions, suggesting a band structure diagram describing a type I heterojunction. The photocatalytic activities calculated demonstrate that this property is related to the surface area and porosity of the samples, the semiconductor nature of the g-C_3_N_4_ structure, and, in this case, the heterojunction that modifies the band structure. These results are of great importance considering that scarce reports are found concerning exfoliated B/S-doped heterojunctions.

## Introduction

Water scarcity is a global issue of concern. More than 80% of globally generated wastewater is discarded without any kind of treatment, polluting water bodies at an alarming rate and causing major ecosystem degradation and biodiversity loss.^1^ One of the major sources of pollution comes from the discharge of synthetic dyes from the textile, cosmetic, leather, and other related industries.^2,3^ It is reported that about 15 000 tons of dyes are released annually into wastewater,^3^ which is extremely harmful due to their toxic, mutagenic, or carcinogenic nature.^4,5^ Even at low concentrations, long-term exposure can cause severe damage to the central nervous system, liver, and kidneys, besides being toxic to aquatic life.^3,6^ Thus, it is of great importance to develop and implement efficient and cost-effective methods for wastewater treatment for the removal of synthetic dyes from water bodies.

Recently, photocatalytic semiconductor technology has emerged as a “green” and sustainable technology for the remediation of environmental organic contaminants such as dyes.^7−9^ Semiconductor photocatalysts use photons with energy greater than the band gap of the material (*hν* > *E*_g_) to excite electrons (e^–^) from the valence band (VB). The excited electron goes to the conduction band (CB), leaving an electron–hole (h^+^) in the valence band.^10^ Electrons in the CB have a chemical potential of 0.5 to −1.5 V versus the normal hydrogen electrode (NHE), and holes in the VB have a chemical potential of 1.0–3.0 V versus NHE, exhibiting a strong reduction and oxidation capacity, respectively. Since the reactions of interest are performed in water, the major electron and hole acceptors are dissolved oxygen and water, respectively.^10−12^ Nevertheless, oxygen reduction occurs at a potential of −0.33 V versus NHE at pH 0 and water oxidation occurs at 2.69 V versus NHE at pH 0, limiting the minimum threshold for the position of the CV and VB for the reaction to occur. Besides this limitation, several catalysts are made of expensive metals; most of them only work under UV light (∼5% of incident sunlight on Earth’s surface), have high rates of electron–hole recombination, possess low surface areas, or lack stability and reusability for prolonged use.^10−12^ Thus, to have a wide and effective implementation of photocatalysis for wastewater treatment, more efficient and stable materials must be developed.

Graphitic carbon nitride (g-C_3_N_4_) is a bidimensional polymeric semiconductor made of repetitive heptazine units. It has been widely studied in recent years due to its low cost and ease of fabrication, property tunability, high thermal and chemical stability, and high photocatalytic response. It is fabricated by thermal polycondensation/polymerization at 500–700 °C of readily available and, in some cases, inexpensive reactants, such as urea, melamine, cyanamide, dicyandiamide, and thiourea.^13,14^ By adding different reactants and varying their concentration during synthesis, the material can be doped with different elements such as O, B, S, Fe, Ni, and Co or creating vacancies of N or C, allowing to tune the band gap of the material and its semiconductor type (n or p).^15,16^ In addition, other systems such as Ag@SnO_2_-g-C_3_N_4_ nanostructures were found to have antibacterial and photocatalytic properties.^17,18^ Usually, the resulting bulk product consists of several stacked sheets of g-C_3_N_4_, limiting its surface area for light absorption and chemical reaction. To overcome this, several processes have been studied to exfoliate the material, including thermal, chemical, thermochemical, acoustic (sonicating), and electrochemical methods or their combinations.^19,20^ The resulting nanosheets display increased surface areas, larger *E*_g_ values, and better photocatalytic performance than their bulk counterparts. To limit the electron–hole recombination in g-C_3_N_4_, the nanosheets have been modified with electron-withdrawing groups, along with the fabrication of homojunctions, heterojunctions with other semiconductors, or Schottky-junctions with plasmonic metals.^21,22^ It has been demonstrated that pure g-C_3_N_4_ has problems associated with insufficient sunlight absorption along with fast electron–hole recombination.^16^ Element doping, such as P,^23−25^ S,^26−29^ O,^30,31^ B,^32^ and N,^33^ among others, appears as a solution that allows the tuning of the electronic structure and band gap.^16^ In this work, we present the synthesis, characterization, and photocatalytic study of heterojunctions involving B- and S-doped g-C_3_N_4_-exfoliated materials. These materials were tested in the degradation of methylene blue, a dye frequently used for coloring plastic, cotton, wool, silk, and jute.^34^

## Experimental Section

### Synthesis of Undoped and Doped g-C_3_N_4_ Samples

Graphitic carbon nitrides were prepared by thermal polycondensation of urea and thiourea.^35^ High-purity reagents used without further treatment, urea 98% (Alfa Aesar), thiourea 99% (Carlo Erba), oxalic acid dihydrate 99.9% (Merck), potassium hydroxide 99% (Merck), boric acid 99.5% (Sigma-Aldrich), and isopropanol 99.5% (Panreac), were used in this work. All g-C_3_N_4_ and derived samples were basically synthesized following the same method, with differences in the reagents used according to the target precursor. In a typical process, different reactants were dissolved in 50 mL of type I water (18 MΩ) and dried overnight at 80 °C. The resulting solid (precursor) was transferred into a semiclosed porcelain crucible, wrapped with aluminum foil, and calcined at 550 °C for 4 h in a muffle furnace with a heating rate of 2.5 °C min^–1^. After the reaction, the crucible was cooled down to room temperature. Then, the product was ground into a fine powder using a mortar and pestle and stored for further use. Following this procedure, bulk unmodified g-C_3_N_4_ (bCN) was prepared by using 20 g of urea. In the case of bulk, sulfur-doped g-C_3_N_4_ (bCN-S), the precursor was fabricated by dissolving 9 g of urea, 11.418 g of thiourea, 5 g of oxalic acid, and 0.12 g of KOH in 75 mL of type I water. In this case, it is expected that the presence of O atoms is introduced into the g-C_3_N_4_ structure by replacing N atoms or by the addition of oxygen-containing surface functional groups (−OH and C=O) under the effect of oxalic acid. Additionally, the use of KOH as an alkali reagent generates N defects on the g-C_3_N_4_ structure due to the reaction of OH^–^ ions with the amine groups produced during thermal polymerization. These reactions result in the formation of N-vacancies and cyano groups (–C≡N) on the surface of the material. Moreover, potassium atoms could be intercalated between the g-C_3_N_4_ layers after the treatment with KOH.^36,37^ Bulk boron-doped g-C_3_N_4_ (bCN-B) was prepared from a mixture of equimolar amounts of urea and thiourea (9 and 11.418 g, respectively) and adding 1% of mass of boric acid to the total mass. Bulk g-C_3_N_4_/g-C_3_N_4_ metal-free heterojunction (bCN-h) was obtained from a mixture of equimolar amounts of urea and thiourea (9 and 11.418 g, respectively).^21^

### Exfoliation of Undoped and Doped g-C_3_N_4_ Samples

Graphitic carbon nitride nanosheets were obtained by thermal exfoliation. In a typical process, 750 mg of bulk g-C_3_N_4_ was placed in an open porcelain crucible and heated at 500 °C for 2 h at a heating rate of 5 °C min^–1^. After the exfoliation, the crucible was cooled down to room temperature, and the samples were ground and then stored for further use. The new exfoliated samples were named eCN-S and eCN-h (from bulk precursors bCN-S and bCN-h, respectively). In the case of bCN and bCN-B, the samples completely evaporated during the exfoliation process, and only the bulk materials were studied.

### Synthesis of Doped g-C_3_N_4_ Heterojunctions

In a typical process, 100 mg (or the amount necessary according to the mass percent of combination) of each type of g-C_3_N_4_ (Table 1) was transferred into a 50 mL beaker with 30 mL of isopropanol. The suspension was sonicated for 1 h to guarantee a homogeneous dispersion of the nanosheets and then heated overnight at 90 °C under constant stirring until complete evaporation of the solvent. After cooling to room temperature, the beaker was transferred to a muffle furnace and heated at 300 °C for 2 h using a heating rate of 5 °C min^–1^. In Table 1, the studied combinations are summarized. In this nomenclature, CN-B/S-*X*%, CN-B/h-*X*%, and CN-h/S-*X*%, *X* corresponds to the mass percent of eCN-S and eCN-h mixed with bCN-B and eCN-h, respectively.

**Table 1:** Doped g-C_3_N_4_ Heterojunctions

	bCN-B	eCN-h
eCN-S	CN-B/S-50%	CN-h/S-25%
		CN-h/S-50%
		CN-h/S-75%
eCN-h	CN-B/h-50%	

After evaluating the photocatalytic performance of the heterojunctions, the combination of eCN-S/eCN-h (CN-h/S-*X*%; *X* = 25, 50, and 75) was chosen for further study due to the enhanced properties. In this sense, heterojunctions with 25, 50, and 75 mass % of eCN-S were prepared using the same procedure as described before.

### Characterization

Powder X-ray diffraction (XRD) patterns were acquired with a Panalytical Empirean diffractometer in a Bragg–Brentano-type focusing geometry, using a Cu Kα_1_ radiation source (λ = 1.540598 Å) and an angular range of 5–100° (2θ). UV–vis diffuse reflectance spectroscopy (UV–vis DRS) was performed with a Specord 50 Plus equipped with an integrating sphere. Spectra were taken using a wavelength step of 1 nm and an acquisition velocity of 5 nm/s in the range from 250 to 1100 nm, using a white Teflon disk as a reference. Reflectance data was transformed to absorbance using the Kubelka–Munk method,^38^ and the band gap was calculated using the Tauc method.^39^ Infrared measurements were carried out in an IRTracer-100 with an ATR module (ATR-FTIR) in the range of 4000–450 cm^–1^. Photoluminescence (PL) experiments were performed in a Cary Eclipse fluorescence spectrophotometer in the range of 400–700 nm using an excitation wavelength of 355 nm. Elemental analysis was performed in a Flash 2000 CHNS-O Analyzer with previously dried samples at 100 °C for 24 h. X-ray photoelectron spectroscopy (XPS) measurements were performed at the Electron Spectroscopy Laboratory (LEe^–^, UFRGS) using a SPECS system equipped with a PHOIBOS 150 1D-DLD hemispherical electron analyzer. An Al Kα (1486.6 eV, 150 W) X-ray source was used, and the base pressure of the analysis chamber was 4 × 10^–10^ mbar. The spectra were analyzed using the CasaXPS software and charge-corrected considering the adventitious carbon signal at a 284.6 eV binding energy. The fitting procedure also addressed the peak line shape asymmetry considering a 20% Gauss–Lorentz ratio as well as a Shirley-type background.

Nitrogen adsorption–desorption isotherms at −196 °C were acquired using the sorption analyzer Autosorb IQ_2_ from Quantachrome. Before analysis, samples were degassed at 200 °C under a high vacuum overnight. Then, samples were analyzed by measuring adsorption equilibrium points between 4.5 × 10^–5^ and 0.993 of *P*/*P*_0_ and desorption equilibrium points in the range of 0.993 and 0.05. BET specific surface area was calculated by applying the Brunauer–Emmett–Teller equation within the range of 0.03–0.3 of *P*/*P*_0_.^40^ Pore size distributions were determined using the Barrett–Joyner–Halenda (BJH) method, applied on the desorption branch.^41^

### Photocatalytic Measurements

To study the photodegradation kinetics of carbon nitrides, 20 mg of catalyst and 50 mL of a 12 ppm methylene blue (MB) solution at pH = 7 were added to a batch reactor with a cooling jacket. The suspension was kept in dark conditions with constant stirring for 30 min to reach absorption/desorption equilibrium. Afterward, the reactor was irradiated for 150 min with a solar simulator from ABET Technologies model 10500, equipped with a 300 W xenon arc lamp, with an irradiance of 100 mW cm^–2^. During the experiment, the reactor temperature was kept constant at 25 °C, and aliquots of 700 μL were taken every 5 min and stored at 1 °C in the dark. Once the photodegradation experiment was finished, all samples were centrifuged at 2800 rpm for 10 min, and 200 μL of the supernatant was transferred to a 96-well microplate. The visible spectrum was measured between 400 and 800 nm using a Multiskan SkyHigh microplate spectrophotometer.

## Results and Discussion

### Characterization of the Samples

**Figure 1:**
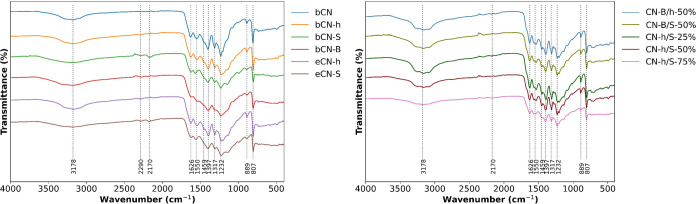
FTIR spectra of undoped, doped, exfoliated, and heterojunctions of g-C_3_N_4_.

Fourier transform infrared (FTIR) spectroscopy was used to characterize all of the samples prepared in this work. The normalized FTIR spectra (Figure 1) display the characteristic peaks of g-C_3_N_4_ and some additional signals associated with functional groups generated by incorporated doping elements. The broad band around 3178 cm^–1^ corresponds to the stretching modes of the hydroxyl groups formed by the oxidation of the carbon nitride during synthesis and uncondensed amine groups at the edge of the nanosheets.^42^ The weak band at 2290 cm^–1^ is associated with adsorbed nitrogen that is released during the polycondensation process, and the small signal at 2170 cm^–1^ for the bCN-S and eCN-S samples is attributed to the asymmetric stretching mode of the cyano groups generated by the addition of KOH in the g-C_3_N_4_ precursor.^7^ The bands between 1700 and 1200 cm^–1^ are associated with the characteristic stretching mode of the C–N bonds of the triazine heterocycle, the small peak at 889 cm^–1^ is attributed to the cross-linking N–H deformation between carbon nitride layers, and the signal at 807 cm^–1^ is assigned to the characteristic vibrational modes of the heptazine units.^22^ Upon exfoliation, there is an increment in the signal’s intensity (unclear in Figure 1 due to normalization) associated with surface hydroxyl groups and amino groups, indicating further oxidation of the material and possibly the formation of more terminal amines from the breaking of stacked nanosheets into smaller pieces. In the case of heterojunctions, the same pattern is observed, indicating that there was no significant change in the structure of the material. The most notable modification is the increase of hydroxyl and terminal amino groups during heterojunction formation, coming from further oxidation of the material during the fabrication of the junction.

**Figure 2:**
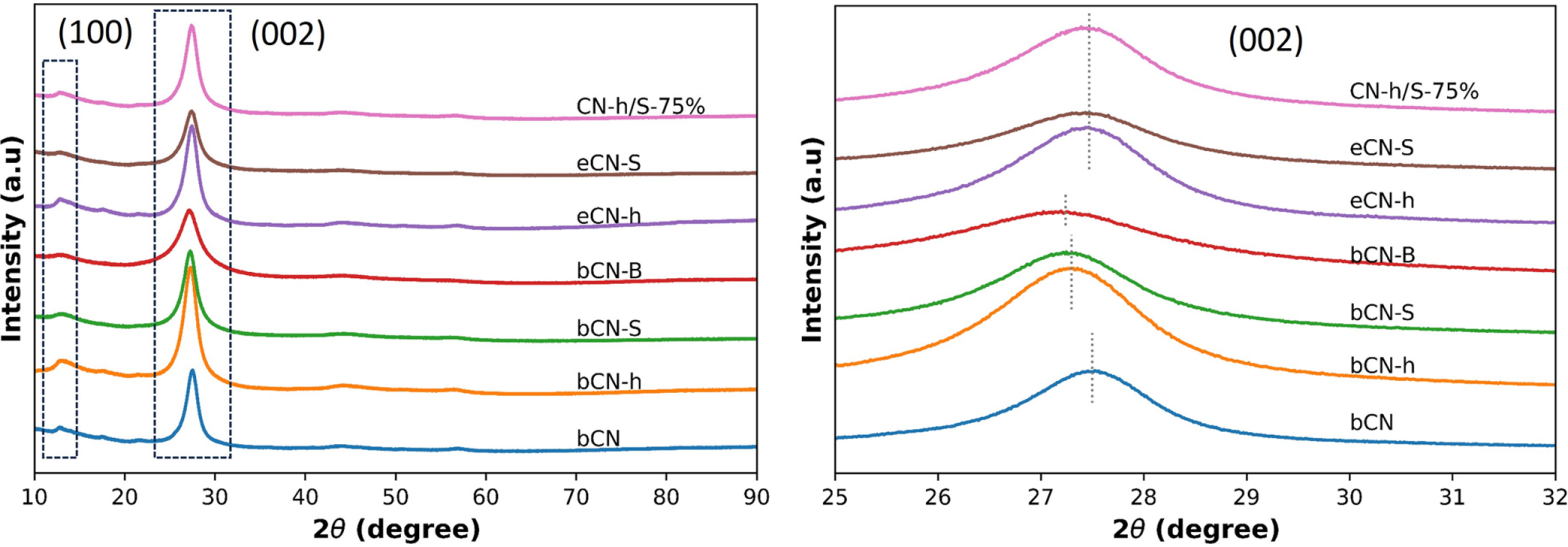
XRD patterns of undoped, doped, exfoliated, and heterojunctions of g-C_3_N_4_.

Diffractograms obtained from X-ray powder diffraction measurements exhibit the characteristic peaks previously observed for g-C_3_N_4_.^21,42−44^ A low-intensity peak between 12 and 13° (2θ) indexed to the (100) planes and a high-intensity peak between 27 and 28° (2θ) corresponding to the (002) planes. The (100) planes, with calculated interplanar distances between 0.6 and 0.7 nm, are associated with the in-plane packaging of repetitive heptazine units. Upon exfoliation, the decrease in intensity of the (100) peak indicates the reduction in the in-plane size of the stacked nanosheets due to loss of monomeric units at the edge of the sheets. The (002) planes correspond to the interlayer stacking of aromatic systems of the different g-C_3_N_4_ nanosheets. In Figure 2, the effect of different precursors and dopants on the stacking behavior of graphitic nanosheets is observed. When only urea is used, a stacking with a separation of 0.3336 nm is obtained; for the samples bCN-h and bCN-S, the distance between planes increases to 0.3358 nm, and upon the addition of boron, the distance increases to 0.3366 nm, which could be related to modifications in the surface of the layers affecting the stacking. After exfoliation, the distance between nanosheets remains constant at 0.3340 nm, indicating a small interlayer spacing, but the intensity of the peaks decreases, indicating a reduction in the number of stacked layers in the material. Thus, upon exfoliation, a slightly more compact structure is obtained but with less nanosheets on it. In Figure 2, only the diffractogram of the CN-h/S-75% composition is presented, considering that the other compositions present similar diffraction patterns.

Elemental analysis from flash combustion (Table 2) corroborates that the oxidation of the g-C_3_N_4_ moiety occurs during the thermal polycondensation process.^44^ In the case of bCN-S, the percentage of oxygen is greater than that of the other materials due to the presence of oxalic acid in the precursor, which reacts with urea during the thermal polycondensation process and decreases the concentration of N–H groups. The presence of a small amount of sulfur indicates that bCN-S, doped with this element, could be affected by the incomplete condensation of urea and thiourea, possibly affected by the presence of oxalic acid or KOH in the reaction. For bCN-h, the absence of sulfur indicates that the homojunction g-C_3_N_4_/g-C_3_N_4_ was formed as the physical appearance of the solid is different from the bCN and no sulfur doping is present. In the case of bCN-B, the percentage of 1.07% boron was calculated by subtracting to 100% the weight percentage of the other elements. After thermal exfoliation, the amount of oxygen increased, indicating further oxidation of the material, as seen in the FTIR spectra. In the case of eCN-S, the amount of sulfur drastically decreased, indicating that this doping element is not strongly embedded in the polymeric matrix. For all cases, the C/N ratio is lower than 0.75. The obtained samples (Table 1) have N-rich compositions probably due to the carbonization of N-rich precursors such as urea.^45^ Nitrogen-rich g-C_3_N_4_ compositions have improved catalytic properties^46^ and are obtained in the presence of solvents such as DMF.^47^ Other strategies are applied to modulate the C/N content due to the demonstrated applications;^48^ however, in this work, such C/N ratio was not controlled on purpose.

**Table 2:** Elemental Analysis from Flash Combustion for Undoped, Doped, and Exfoliated g-C_3_N_4_

sample	C, wt %	N, wt %	H, wt %	O, wt %	S, wt %	B, wt %	C/N ratio
bCN	34.25	58.07	1.97	5.70	0	0	0.69
bCN-h	34.11	59.83	1.85	4.21	0	0	0.66
bCN-S	33.82	57.12	1.55	6.76	0.75	0	0.69
bCN-B	32.22	59.73	2.03	5.00	0	1.07	0.63
eCN-h	34.08	50.81	1.91	6.19	0	0	0.69
eCN-S	33.98	54.99	1.44	10.23	0.36	0	0.70

**Figure 3:**
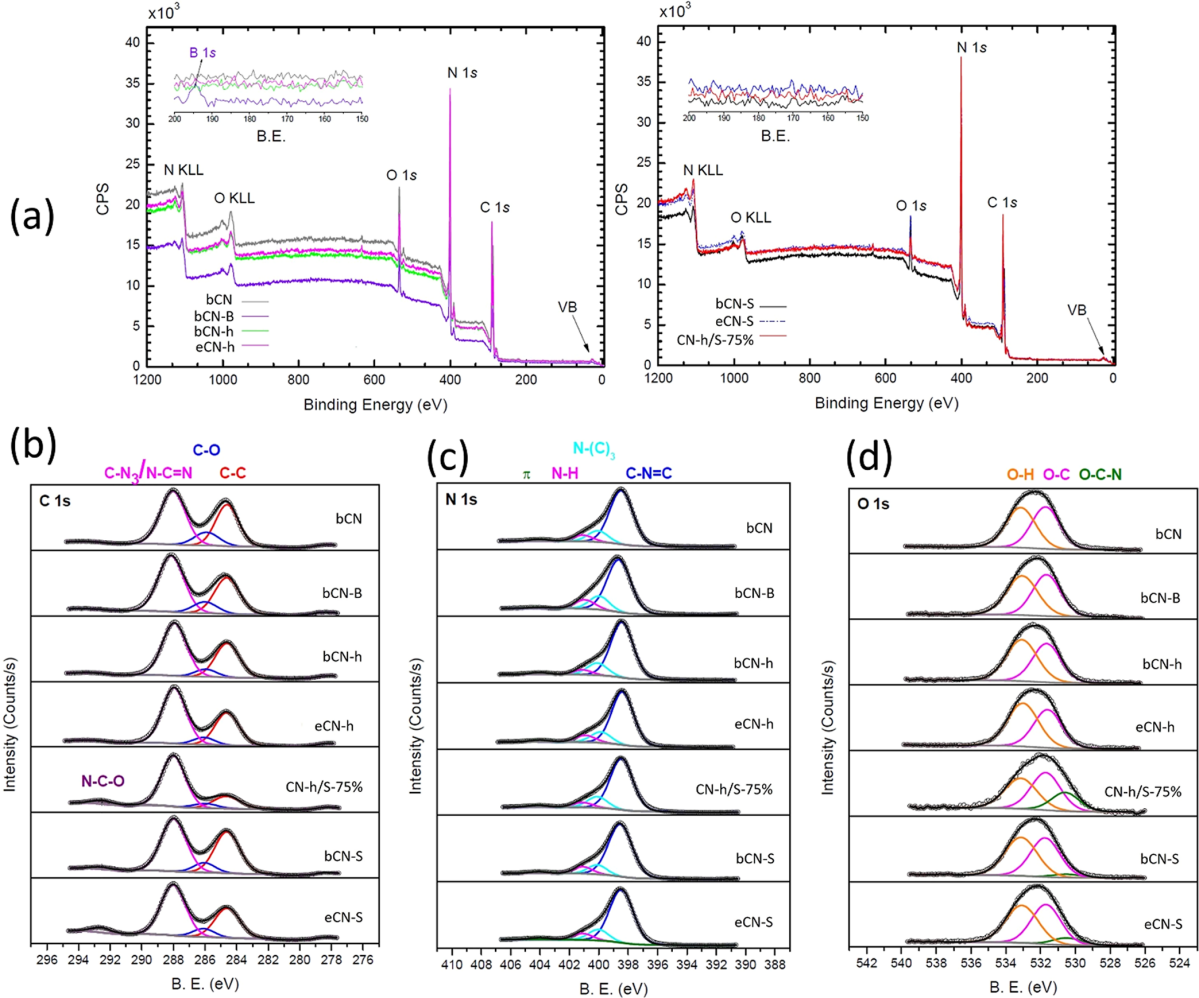
XPS spectra, (a) survey (insets between 200 and 150 eV are shown), (b) C 1s, (c) N 1s, and (d) O 1s for undoped, doped, exfoliated, and heterojunctions of g-C_3_N_4_.

XPS surface chemical analyses were used to probe the outermost atoms present at the samples’ surface as well as their chemical states. Figure 3a displays the full survey spectra, indicating the corresponding C 1s, N 1s, and O 1s photoemission peaks and the corresponding Auger signals. These main elements are present in all samples, while a faint boron XPS signal was detected in the bCN-B sample, but no sulfur signal in the bCN-S and eCN-S was noticeable (see the inset of Figure 3a). This is probably due to the low surface concentrations, which are below the detection limit of the technique,^49^ considering that low amounts of sulfur were previously detected in the elemental analysis from flash combustion, a more sensitive and suitable technique.

Table S1 displays the surface atomic composition as determined from the XPS experiments. The calculated C/N ratios are also listed, and it is observed that samples bCN and bCN-S present ratio values quite close to the expected 1.50. On the other hand, sample CN-h/S-75% has the highest relative nitrogen concentration.

The high-resolution C 1s XPS spectra (Figure 3b) were deconvoluted to determine the carbon chemical components and are in accordance with literature reports.^15,19−21,35,41^ Three chemical components were determined for bCN, bCN-h, eCN-h, and bCN-B located around 248.6, 285.9, and 288.0 eV ascribed to adventitious or graphitic carbon (C–C), C–O, and N=C–N (or C–N_3_) bonds, respectively. An additional high-energy feature is observed around 292.5 eV for samples bCN-S, eCN-S, and CN-h/S related to N–C–O bonds. The high-resolution N 1s XPS spectra (Figure 3c) were deconvoluted with four nitrogen chemical environments: C–N=C, N–C3, and N–H bonds and a high-energy π transition. The high-resolution O 1s XPS spectra (Figure 3d) were deconvoluted with two or three components depending on the sample, which correspond to O–C–N, O–C, and O–H bonds.^21^ Again, samples bCN-S, eCN-S, and CN-h/S present the same additional component, O–C–N, corroborating the C 1s analyses. The changes in the surface chemical states for those samples may ultimately impact their photocatalytic performance.

**Figure 4:**
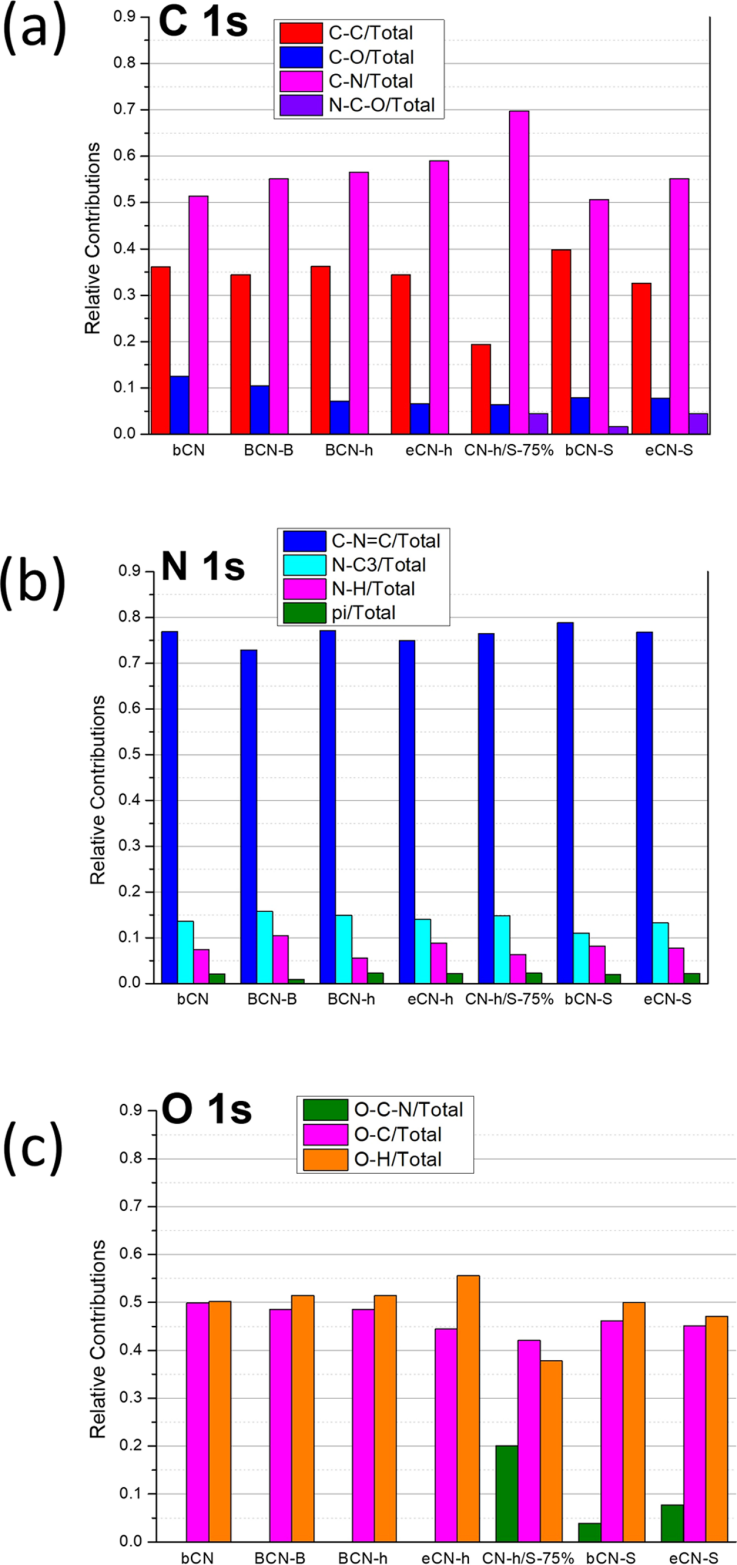
Relative contribution of each chemical component obtained from the fittings of the (a) C 1s, (b) N 1s, and (c) O 1s high-resolution XPS spectra of all samples.

Figure 4 shows the relative contribution of each chemical component obtained from the fittings of the C 1s, N 1s, and O 1s high-resolution XPS spectra of all samples (shown in Figure 3a–c). The area of each component was divided by the total area of the corresponding XPS peak. It enables us to compare and evaluate the chemical bond modifications promoted in each sample preparation. If the relative chemical contributions are compared with samples of bCN, it is possible to observe that most significant changes occur in the C 1s and O 1s regions. Indeed, samples submitted to S treatment (bCN-h/S-75%, bCN-S, and CN-S) promoted the formation of a new component, N–C–O in both peaks. Particularly, the sample bCN-h/S-75% stands out, clearly demonstrating the enhancement of the C–N bonds and reduction of the C–C and C–O bonds. The same trend but less intense is observed for all samples.

### Photocatalytic Activity

**Figure 5:**
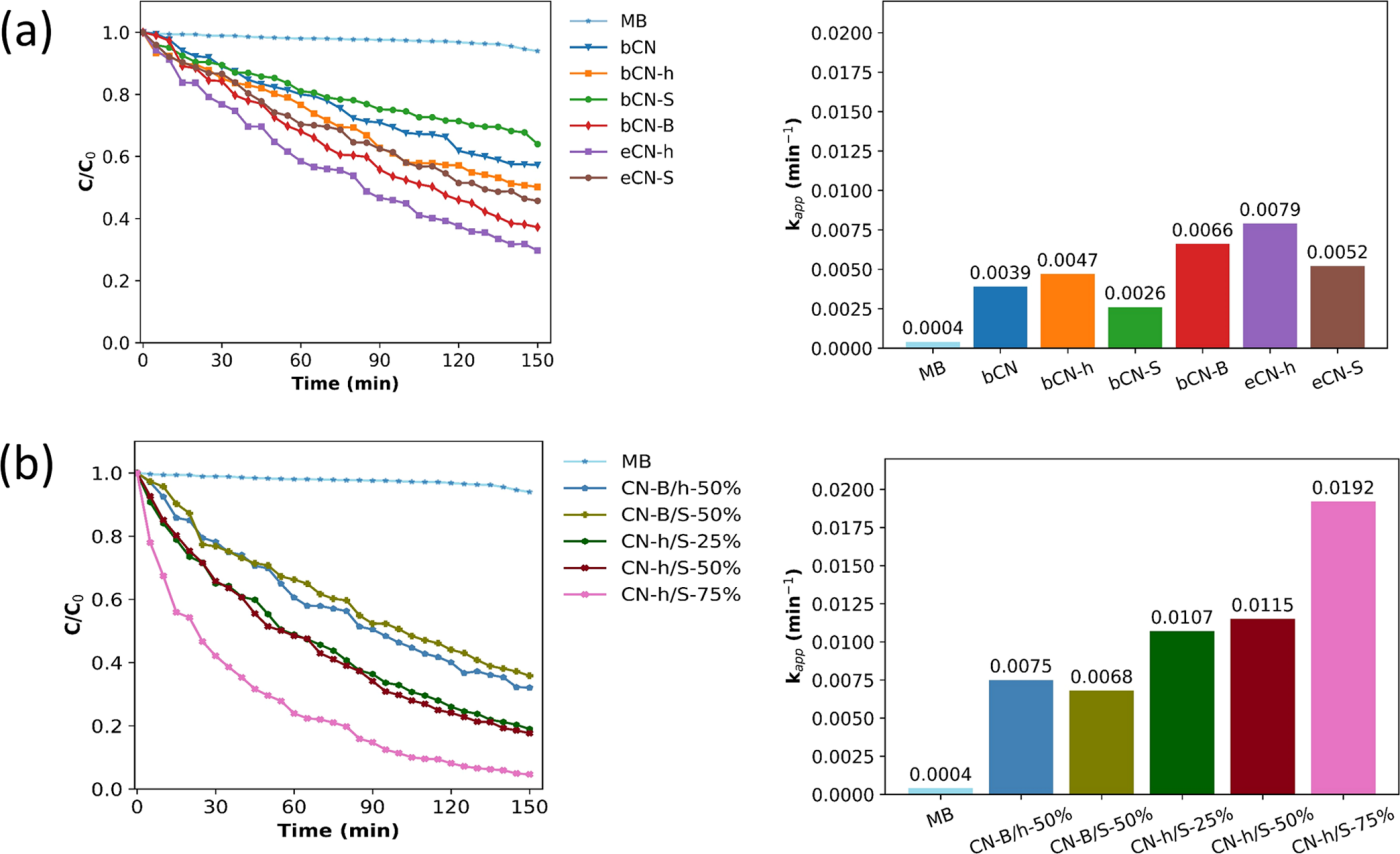
Photocatalytic degradation of methylene blue (MB) and apparent reaction rate constant (*k*_app_) of (a) pristine (bCN), bulk heterojunction (bCN-h), bulk doped (bCN-S, bCN-B), and exfoliated (eCN-h, eCN-S) samples; (b) heterojunctions at different wt % concentrations.

The photocatalytic activity of all synthesized samples was measured by the degradation of methylene blue (MB) under visible radiation using a solar simulator. Before any measurement, the system was allowed to reach absorption/desorption equilibrium by stirring for 30 min under dark conditions.^35^Figure 5 shows the variation of MB concentration (C/C %) as a function of time (min). For nonexfoliated samples, the one doped with boron displayed the best photocatalytic activity by degrading around 60% of the pollutant after 150 min of irradiation, while the bCN-S sample had the worst performance, even below the pristine carbon nitride bCN. Upon exfoliation, the photocatalytic performance of the samples improved, with eCN-h being the most active photocatalyst of all, degrading 70% of all MB after 150 min. Kinetic experiment data were adjusted to a pseudo-first-order reaction model (ln (*C*/*C*_0_) = −*k*_app_*t*), which was used to calculate the apparent reaction rate constant (*k*_app_) (see the correlation coefficients of all samples in the Supporting Information).^48,50,51^ From Figure 5, eCN-h had the best performance with a *k*_app_ of 7.9 × 10^–3^ min^–1^, followed by bCN-B with a *k*_app_ of 6.6 × 10^–3^ min^–1^. In the case of the heterojunctions, the first to be fabricated were CN-B/h-50%, CN-B/S-50%, and CN-h/S-50%. The samples containing the boron-doped material did not show a significant improvement in the photocatalytic activity of the heterojunction, and in the case of CN-B/h-50%, the results were even worse than those of the eCN-h sample. Only the combination of eCN-h with eCN-S displayed a higher rate of MB degradation than that of its constituting components, making it a good candidate for further evaluation. Thus, two other heterojunctions with these samples were fabricated by using different proportions. From the results (Figure 5), it is evident that upon the increase of eCN-S on the heterojunction, the photocatalytic performance increases, the sample with 75% of this graphitic (CN-h/S-75%) being the one with the higher *k*_app_ with a value of 1.92 × 10^–2^ min^–1^ and degrading almost all of the MB in the solution at 150 min. The observed *k*_app_ value is of considerable importance and suggests a worthy photocatalytic activity compared to the literature. The *k*_app_ value reported for MB degradation by the tubular g-C_3_N_4_/carbon framework is 1.32 × 10^–2^ min^–1^ using visible light irradiation by a Xe lamp, which corresponds to similar conditions used in this work.^51^ Other reports present higher *k*_app_ values; however, the experimental conditions and the dye used are not directly comparable.

### Optical Characterization

**Figure 6:**
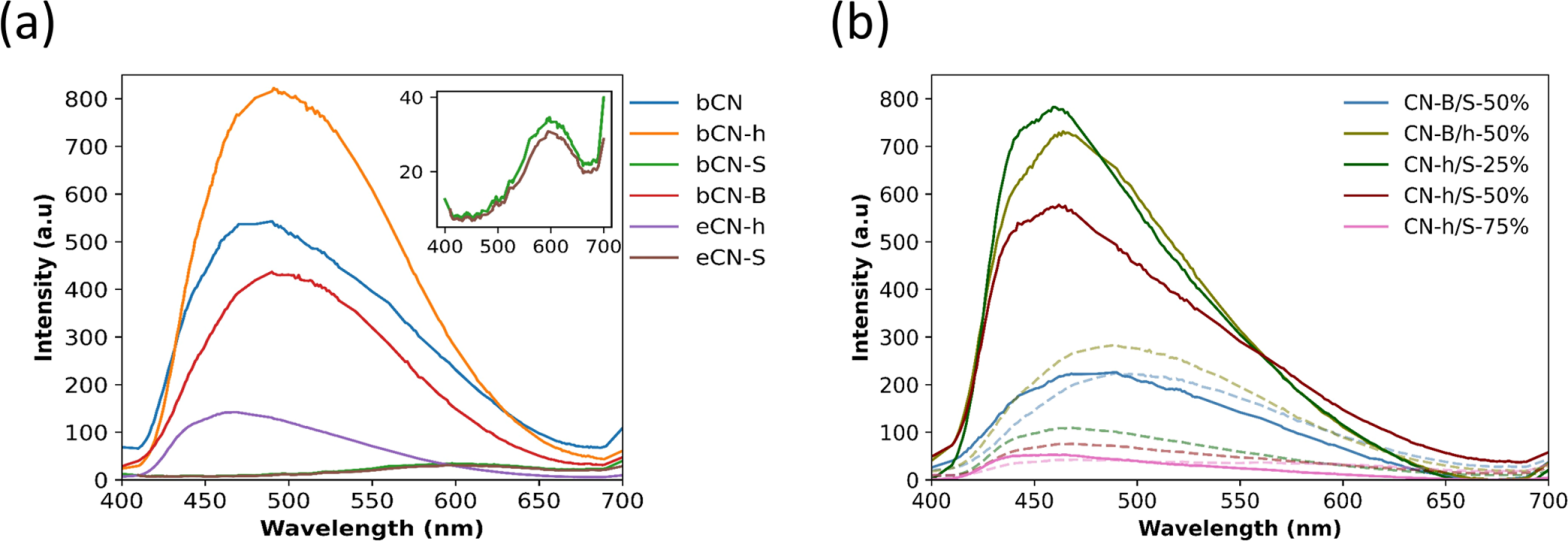
Room-temperature photoluminescence (PL) spectra for (a) pristine (bCN), bulk heterojunction (bCN-h), bulk doped (bCN-S, bCN-B), and exfoliated (eCN-h, eCN-S) samples and (b) heterojunctions at different wt % concentrations. The inset between 400 and 700 nm is shown in panel (a) for clarity.

To further understand the performance of the samples and the heterojunctions, photoluminescence (PL) experiments were performed, along with UV–vis diffuse reflectance and VB-XPS. From PL, it is possible to obtain information regarding the migration, transfer, and separation of photogenerated charge carriers.^21^ The information obtained from this technique allows the measurement of emissions that are proportional to the radiative recombination of electron–hole pairs. Figure 6 shows that the samples present the same spectroscopic tendency displayed by broad luminescent bands. In the case of the pristine bCN sample, the band can be centered at around 470–480 nm. However, the corresponding bCN-h presents a shift toward higher values (≈500 nm). Additionally, the intensity of the emission increased compared to bCN, which denotes that this sample presents an increase in electron–hole recombination. However, the exfoliated eCN-h heterojunction presents a recombination of higher energy (≈460 nm), and the probability of this recombination decreases considerably. The doped sample bCN-B does not present better conditions as a photocatalyst compared to the enhanced properties of the eCN-h due to the high intensity of emission. Figure 6 shows that the best conditions for photocatalytic applications are observed for bCN-S and eCN-S with the lowest intensity of emission. Following this analogy, from PL measurements, it is possible to characterize the CN-h/S-75% heterojunction as the best candidate for photocatalytic applications due to the less probable electron–hole recombination compared to the other heterojunctions. At this point of the discussion, eCN-h and CN-h/S-75% heterojunctions have the best conditions for MB degradations under photoexcitation. However, the energy of recombination represents an important factor for the application due to its correlation with the band gap of the materials. The best photocatalytic degradation of methylene blue (MB) and the higher apparent reaction rate constant (*k*_app_) were measured for CN-h/S-75%.

**Figure 7:**
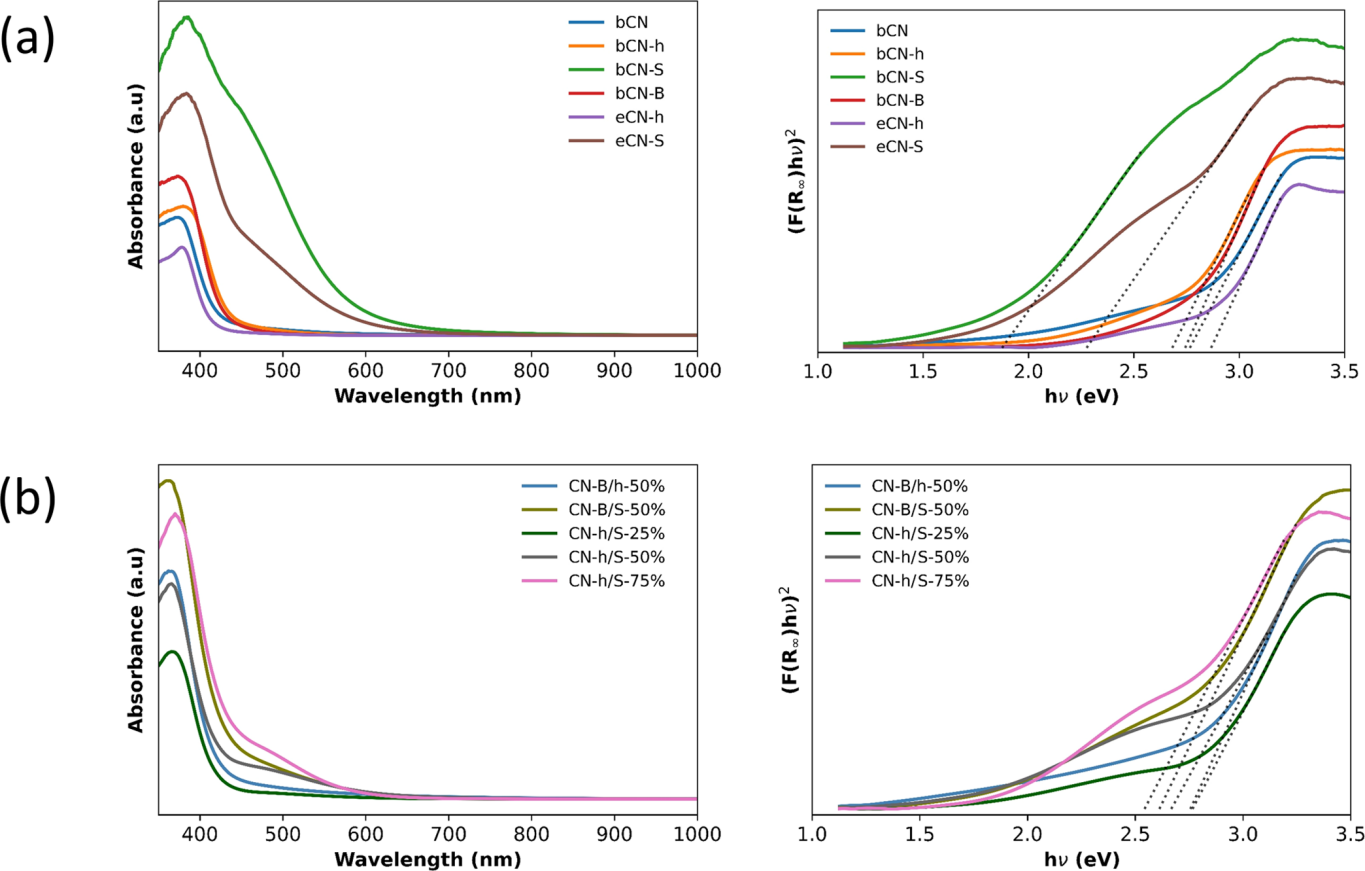
UV–vis diffuse reflectance (left) and Tauc [(α*h*ν)^1/γ^; γ = 2 for indirect transition] (right) plots for (a) pristine (bCN), bulk heterojunction (bCN-h), bulk doped (bCN-S, bCN-B), and exfoliated (eCN-h, eCN-S) samples and (b) heterojunctions at different wt % concentrations.

In order to understand the meaning of the PL results and their correlation with the photocatalytic activity, UV–vis diffuse reflectance (see raw spectra in the Supporting Information) was used to determine the absorption edge of the synthesized materials (Figure 7). To calculate the band gap of the materials, the Tauc method was used, the band-gap energy being estimated from the intercept of the tangent calculated in the plots of (α*h*ν)^1/2^ versus photon energy.^39^ Previously to the use of this model, the Kubelka–Munk function was applied to transform to absorbance the diffuse reflectance data (Figure 7).^39^

The pristine graphitic structure bCN has a band gap of 2.8 eV, which is reduced upon the incorporation of doping elements. In the case of bCN-h, the band gap is 2.7 eV; for bCN-S, it is 1.9 eV; and for bCN-B, it is 2.7 eV. When exfoliated, the band gap of the samples increases to 2.9 eV for eCN-h and 2.3 eV for eCN-S due to quantum confinement effects caused by the reduction of the nanosheet size. In the case of heterojunctions, the absorption edge of the new materials depends on the amount of eCN-S in the sample; the larger the quantity, the lesser the band gap of the system. For the sample CN-B/h, the band gap is 2.8 eV; for CN-B/S, it is 2.6 eV; for CN-h/S-25%, it is 2.8 eV, for CN-h/S-50%, it is 2.7 eV; and for CN-h/S-75%, it is 2.5 eV. In theory, the measured band gaps are in the visible spectrum, 1.6 eV (red) to 3.2 eV (violet). However, the best electron–hole separation is observed for bCN-h and CN-h/S-75% heterojunctions. The band-gap values are sensible to the sort of g-C_3_N_4_ sample prepared, doped, undoped, bulk, or exfoliated, and obviously the type of heterojunction synthesized. This spectrum of variability demonstrates the great importance of this material in the potential to modulate systems with desired optical properties.

**Figure 8:**
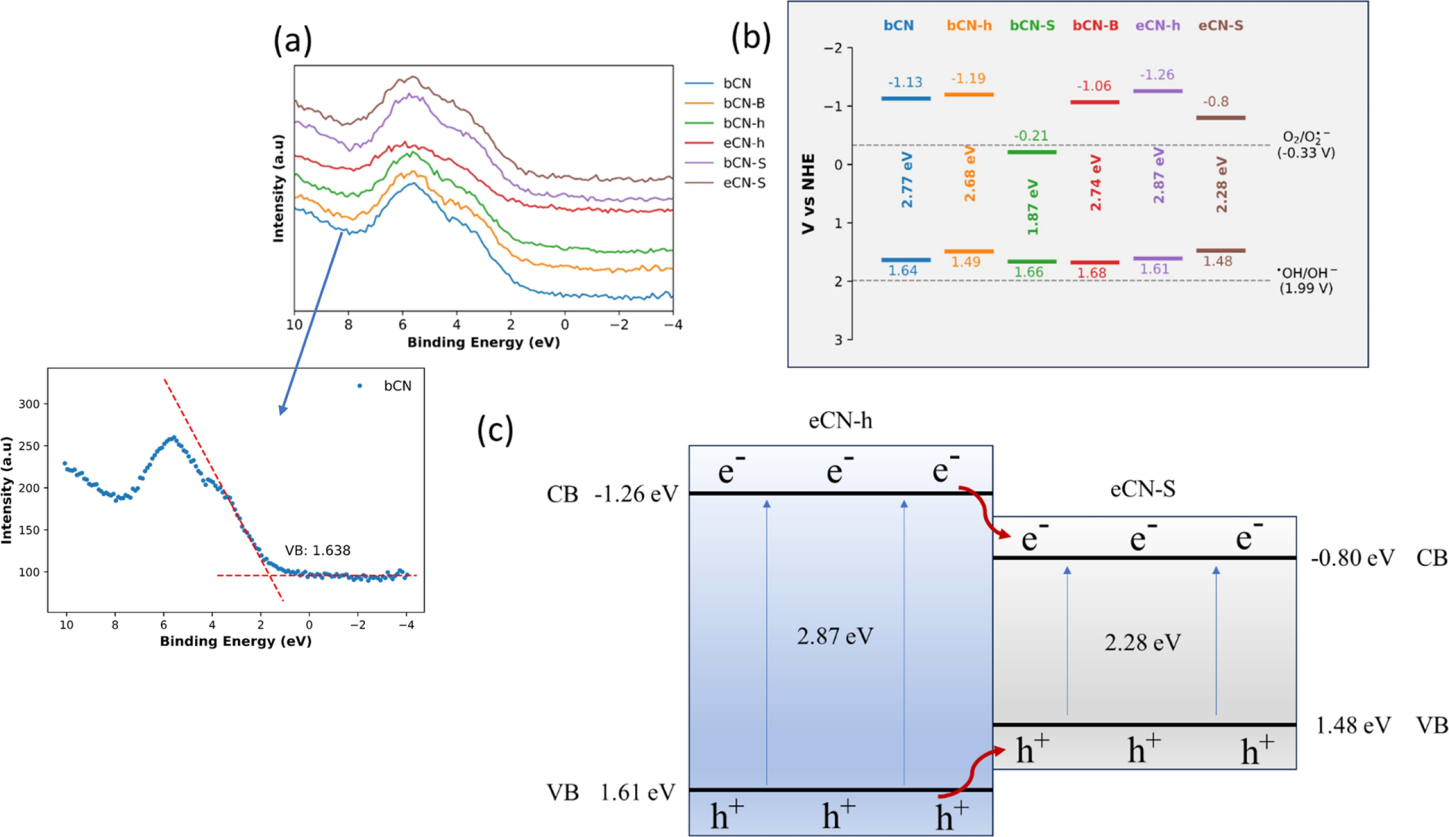
(a) VB-XPS spectra with an example of VB position determination and (b) schematic illustration of the calculated band gap compared to a normal hydrogen electrode (NHE). All valence band determinations are shown in the Supporting Information. (c) Schematic illustration of electron–hole separation at the exfoliated eCN-h/eCN-S heterojunction.

To explore the possible charge-transfer pathways for pollutant degradation, VB-XPS was used to calculate the valence band position of all graphitic samples (Figure 8; see the graphical observation of the valence band determination in the Supporting Information) and then in combination with the measured band gap determine the electronic band structure of the materials. From Figure 8, it is possible to observe that bCN-B has the more positive VB (1.68 eV) while eCN-h has the more negative CB (−1.26 eV). Considering the results obtained from the photocatalytic activity experiments, the highest values of the apparent reaction rate constant (*k*_app_) were obtained for CN-h/S-25% (1.07 × 10^–2^ min^–1^), CN-h/S-50% (1.15 × 10^–2^ min^–1^), and CN-h/S-75% (1.92 × 10^–2^ min^–1^) heterojunctions. These samples represent heterojunctions between eCN-h and eCN-S being 25, 50, and 75 the w% of eCN-S. It is interesting that the *k*_app_ improves with the eCN-S content, but in the pure eCN-S sample, the *k*_app_ value decreases drastically to 5.2 × 10^–3^ min^–1^, while the *k*_app_ value for pure eCN-h is 7.9 × 10^–3^ min^–1^.

These results demonstrate that effectively, for this case, the heterojunction is important to the photocatalytic activity. Based on the VB and CB levels, of eCN-h and eCN-S, the band structure diagram for the eCN-h/eCN-S heterojunction is proposed in Figure 8. The improved photocatalytic performance adjusts with a type I heterojunction.^52^ This sort of heterojunction is not commonly reported for these systems. Type II heterojunctions were reported for g-C_3_N_4_/g-C_3_N_4_ isotype heterojunctions prepared from urea and thiourea.^21,44^ However, an S-scheme was reported for the g-C_3_N_4_/g-C_3_N_4_ isotype heterojunction obtained from hydrothermally treated melamine and urea.^42^ Similarly, to the present work, a type I heterojunction was reported for the intersection between two different samples, the first one prepared by solvothermal reaction using the dicyandiamide as a precursor and the second one was prepared by direct heating from a melamine precursor.^43^

### Surface Area and Porosity Characterization

**Figure 9:**
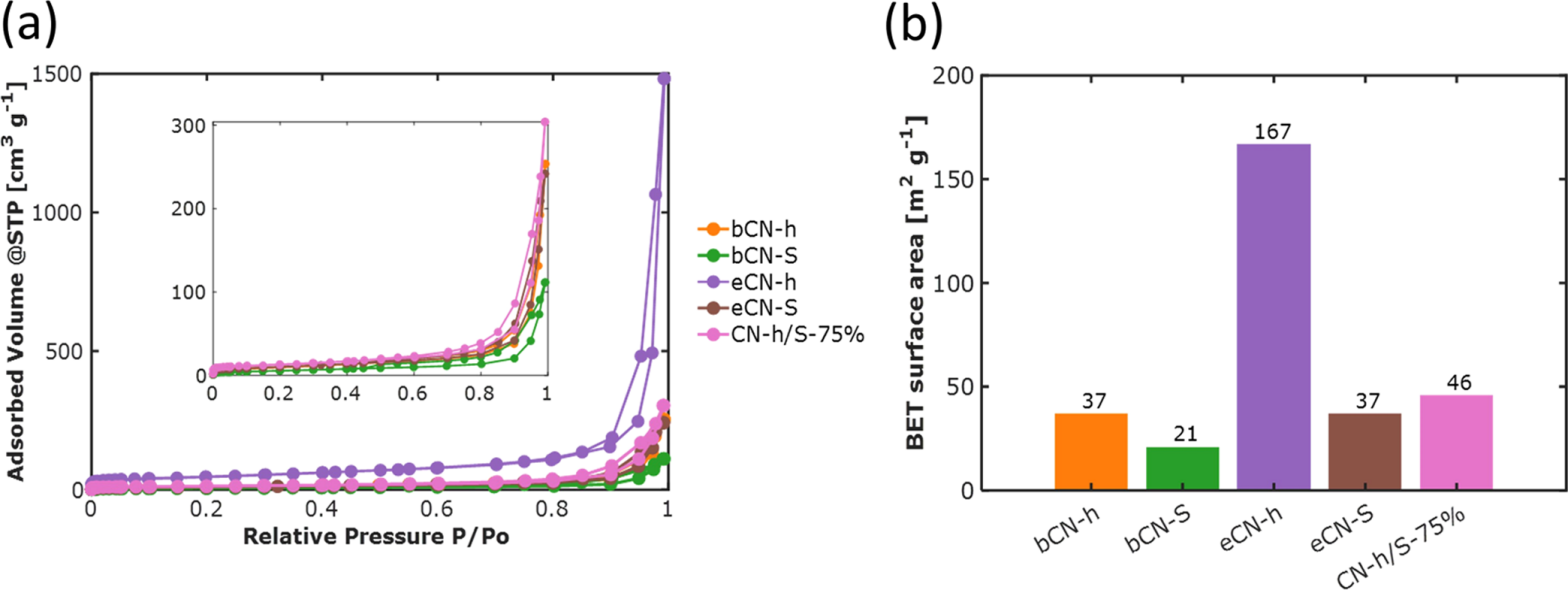
(a) Nitrogen adsorption–desorption isotherms at −196 °C of bulk bCN-h, bCN-S, exfoliated eCN-h, eCN-S, and heterojunction CN-h/S-75% samples; in the inset plot, a closer view of the samples bCN-h, bCN-S, eCN-s, and CN-h/S-75% is presented. (b) BET surface areas for the same five samples.

Figure 9 illustrates the nitrogen adsorption–desorption isotherms and the BET specific surface areas. According to the IUPAC classification, all of the isotherms exhibit type IIb behavior, which signifies the presence of slit-shaped pores.^53^ Furthermore, an H3-type hysteresis loop is observed in all of the isotherms, indicating the existence of mesopores (2–50 nm) formed by nonrigid aggregates of plate-like particles.^54^ The porosity of these materials can be attributed to the interspace, overlapping, and agglomeration of g-C_3_N_4_ nanosheets, which is in line with the characteristic shape of the isotherms.^55^ Comparing the nitrogen adsorbed amount, the exfoliated sample eCN-h presented the highest values, which in turn resulted in the highest surface area.

The BET surface areas of the bCN-h and eCN-h samples were determined to be 37 and 167 m^2^ g^–1^, respectively. This confirms that the exfoliation of the bulk material was successfully achieved with the thermal treatment process, resulting in an increased separation between the aggregates of nanosheet structures and a consequent enhancement in the accessible surface area.^56^ In contrast, the bulk bCN-S and its exfoliated counterpart, eCN-S, exhibited surface areas of 21 and 37 m^2^ g^–1^, respectively. This indicates that although exfoliation led to an increase in surface area, it was not as significant as observed in the latter case. On the other hand, the heterojunction CN-h/S-75% presented a surface area of 46 m^2^ g^–1^ consistent with the earlier findings, as it falls within the range of surface area observed for the eCN-h and eCN-S samples (167 and 37 m^2^ g^–1^, respectively). Furthermore, it is noteworthy that the surface area of the heterojunction CN-h/S-75% resembles the surface area of eCN-S to a greater extent than that of eCN-h, primarily due to the higher percentage of exfoliated sulfur-doped g-C_3_N_4_ used during the synthesis.

**Figure 10:**
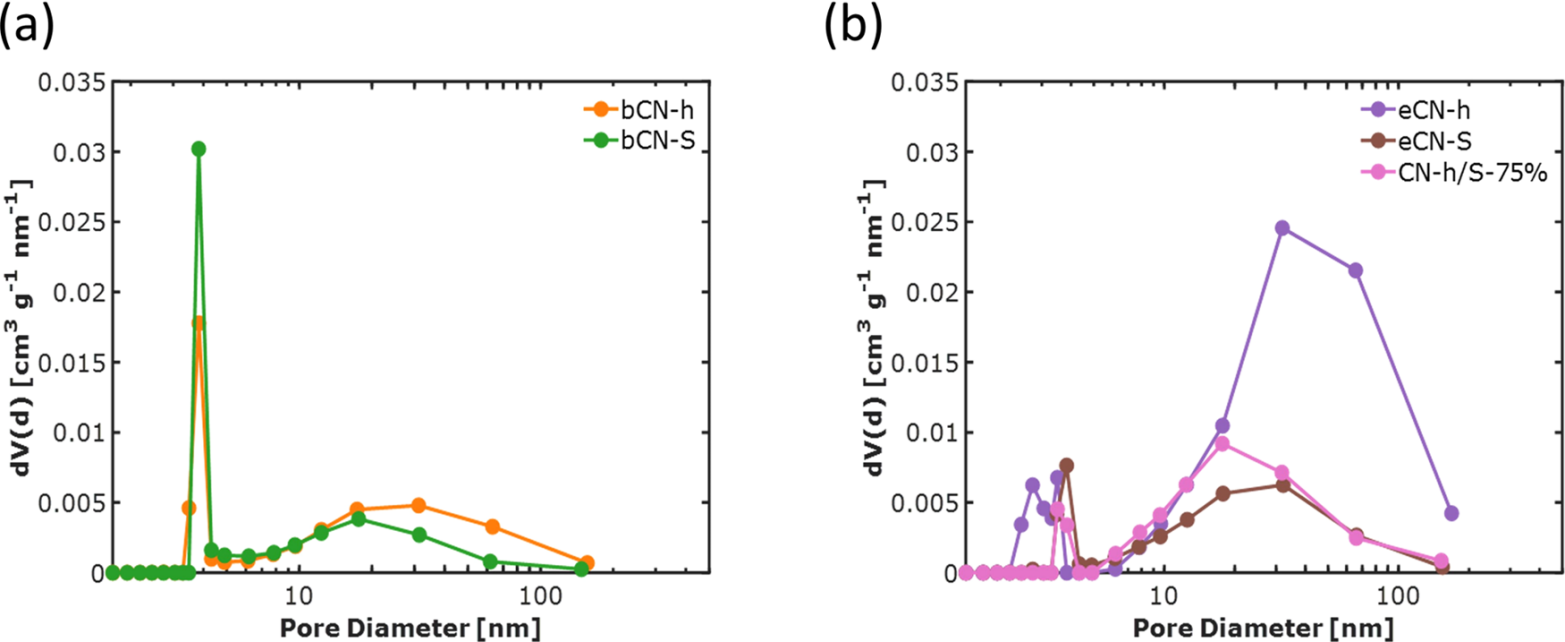
Pore size distributions of (a) bulk samples bCN-h and bCN-S and (b) exfoliated eCN-h and eCN-S and heterojunction CN-h/S-75%.

As previously stated, all of the samples showed a distinctive isotherm shape that is indicative of mesoporosity. This observation is substantiated by the determination of the pore size distribution, which reveals the presence of pores ranging from 2 to 50 nm, as shown in Figure 10. Moreover, the PSD also showed the presence of macroporosity, characterized by pore sizes exceeding 50 nm.^54^ The bulk materials exhibit a narrow distribution of small mesopores, measuring 3.8 nm in width, and a broader range of larger mesopores, with modes at 20 and 30 nm for bCN-S and bCN-h, respectively. In contrast, after exfoliation, a noticeable reduction in the presence of smaller mesopores was observed, along with an increase in the occurrence of wider mesopores. Regarding the heterojunction sample, the quantity of mesopores with a broader width (ranging from 10 to 50 nm) lies between the quantities of such mesopore sizes found in the precursor materials (eCN-h and eCN-S). The determination of the PSD is essential to ensure that the target molecule can effectively access the internal surface area of a material. In the specific case of MB with molecular dimensions of 1.26 × 0.77 × 0.65 nm^3^,^57^ the pore sizes of the resulting g-C_3_N_4_ materials are sufficiently large to allow the entry of this molecule. Consequently, this favorable porosity enables easier diffusion of MB into the pores and enhances its interaction with the surface catalytically active sites.

When designing and investigating photocatalytic systems, it is imperative to consider the textural properties of the photocatalysts, particularly the surface area. This is crucial in the context of heterogeneous photocatalysis because it entails a photochemical reaction that occurs on a solid surface.^58^ This principle also holds true for graphitic carbon nitride materials, as previous research has established that the photoactivity of g-C_3_N_4_ is largely influenced by the abundance of reactive sites provided through high surface areas.^59^ The findings of this study showed a strong correlation between the surface area and photoactivity of the g-C_3_N_4_ samples. For instance, when comparing the bulk samples, bCN-h and bCN-S, it was observed that the best photocatalytic performance was obtained for bCN-h, as well as the greatest surface area. Similarly, the exfoliated samples, eCN-h and eCN-S, followed the same trend, with eCN-h exhibiting greater photoactivity due to its larger surface area.

As mentioned above, the exfoliation process increased the surface area of the bulk materials, thereby enhancing their photocatalytic activity. In the case of the metal-free heterojunction bCN-h and the exfoliated sample eCN-h, the BET area experienced a 4.5-fold increment after exfoliation, while the rate constant (*k*_app_) exhibited a 1.8-fold increase. Conversely, in the case of eCN-S, its surface area was found to be 2.0 times higher than that of its bulk counterpart (bCN-S), accompanied by a kinetic constant that was also twice as high as that of bCN-S. These results suggest that the influence of the surface area on the photocatalytic activity may vary depending on the distinctive characteristics exhibited by a particular sample. Specifically, the bCN-S and eCN-S share the same charge recombination behavior, whereas bCN-h and eCN-h differ in this property, which may account for the observed differences in the effect of the surface area on the photocatalytic performance. Additionally, XPS results for samples bCN-S and eCN-S also presented changes in the electronic structure since additional surface chemical components related to the C–N–O bond formation.

The heterojunction CN-h/S-75% presented a surface area of 46 m^2^ g^–1^, which was at least 3.6 times smaller than that of eCN-h. Despite this, the heterojunction demonstrated a superior capacity for MB photocatalytic degradation compared with the other samples. This finding suggests the presence of a synergistic electronic effect between eCN-h and eCN-S, particularly at higher concentrations of the latter but not by itself. The observed synergy exerts a more pronounced impact on the photocatalytic activity than on the surface area, as demonstrated by the comparison with eCN-h. Again, CN-h/S-75% displayed the most noticeable changes in the electronic structure in both the O 1s and C 1s XPS regions. These observations emphasize that photoactivity is not solely dependent on surface area and highlight the critical importance of considering electronic and optic features such as band gap, electron–hole separation, light absorbance, and electron–hole recombination when evaluating photocatalytic performance.^60^

## Conclusions

Graphitic carbon nitrides and exfoliated B/S-doped heterojunctions were prepared by thermal polycondensation, followed by thermal exfoliation before the thermal formation of the heterojunctions. The low cost of the precursors and the easy fabrication propose a sustainable alternative of remediation to the discharge of synthetic dyes that are released annually in our environment. Exfoliated undoped/S-doped heterojunction with a 75 mass % of S-doped (g-C_3_N_4_) presented an apparent reaction rate constant (*k*_app_) of 1.92 × 10^–2^ min^–1^ exhibiting the best photocatalytic property compared to other compositions studied in this work. This enhanced activity is due to a combination of the surface area and porosity and the induced type I band structure caused by the heterojunction.

## Supporting Information Available

Correlation coefficients for kinetic experiments adjusted to a pseudo-first-order reaction model (ln (*C*/*C*_0_) = −*k*_app_*t*); raw spectra for UV–vis diffuse reflectance measurements; and graphical observation of the valence band determination using VB-XPS (PDF)
