# Supplementary material for: Sulfur-Doped g-C3N4 Heterojunctions for Efficient Visible Light Degradation of Methylene Blue
Source: ACS Omega. 2023 Dec 5;8(50):47821–34. doi: 10.1021/acsomega.3c06320 (PMC10734029; doi:10.1021/acsomega.3c06320)
Supplement: Supplementary file 1 — ao3c06320_si_001.pdf [file ao3c06320_si_001.pdf]

Supporting information

Sulfur doped g-C_3N_4 heterojunctions for efficient visible light degradation of methylene blue

*Andrés F. Pérez-Torres[a], Diego F. Hernández-Barreto[b], Valentina Bernal[b], Liliana Giraldo[c], Juan Carlos Moreno-Piraján[b], Edjan Alves da Silva[d], Maria do Carmo Martins Alves[e], Jonder Morais[d], Yenny Hernandez[f], María T. Cortés[g], Mario A. Macías[a]**

[a] Crystallography and Chemistry of Materials, CrisQuimMat, Department of Chemistry, Universidad de los Andes, Bogotá D.C. 111711, Colombia.

[b] Facultad de Ciencias, Departamento de Química, Grupo de Investigación en Sólidos Porosos y Calorimetría, Universidad de los Andes, Bogotá D.C. 111711, Colombia.

[c] Facultad de Ciencias, Departamento de Química, Grupo de Calorimetría, Universidad Nacional de Colombia, Sede Bogotá 01, Colombia.

[d] Electron Spectroscopy Lab (LEe-), Instituto de Física, Universidade Federal do Rio Grande do Sul (UFRGS), Av. Bento Gonçalves, 9500, 91501-970 Porto Alegre, RS Brazil.

[e] Instituto de Química, Universidade Federal do Rio Grande do Sul (UFRGS), Av. Bento Gonçalves, 9500, 91501-970 Porto Alegre, RS Brazil.

[f] Department of Physics, Universidad de los Andes, Bogotá D.C. 111711, Colombia.

[g] Departamento de Química, Universidad de los Andes, Bogotá D.C. 111711, Colombia.

Diffuse reflectance

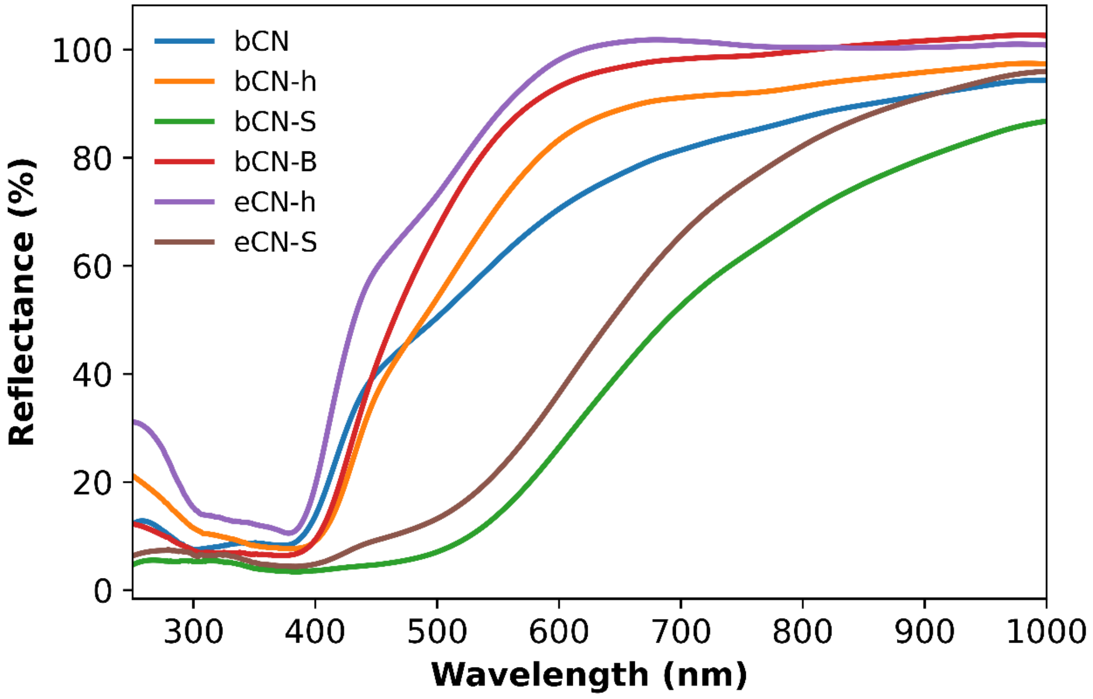

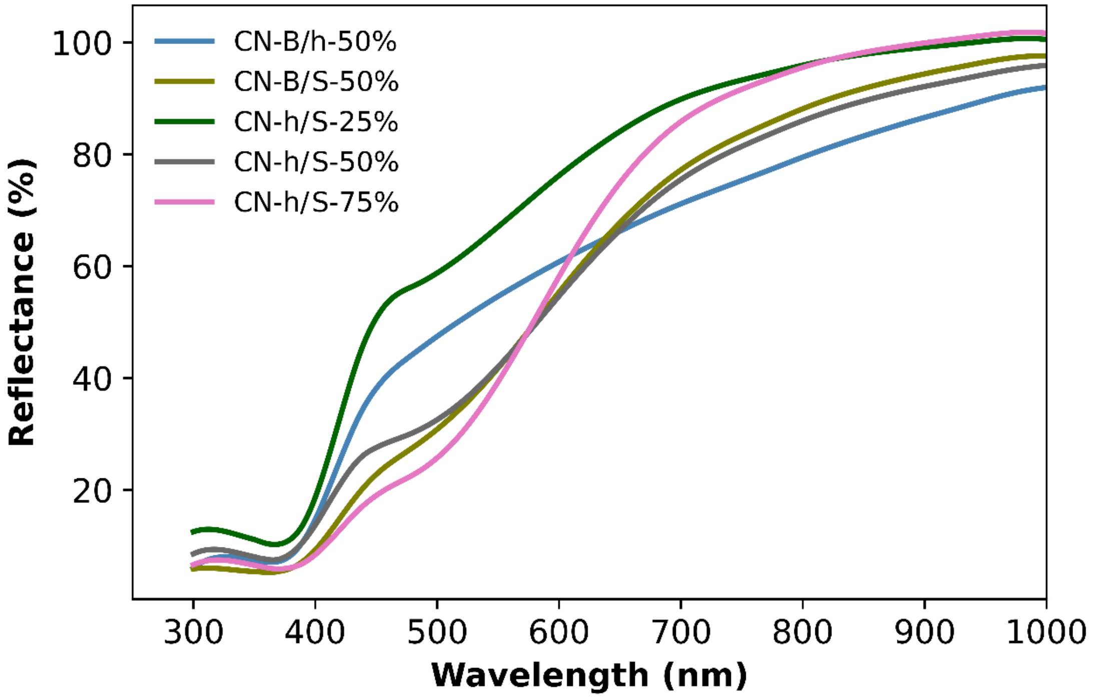

Kinetics

- MB photolysis

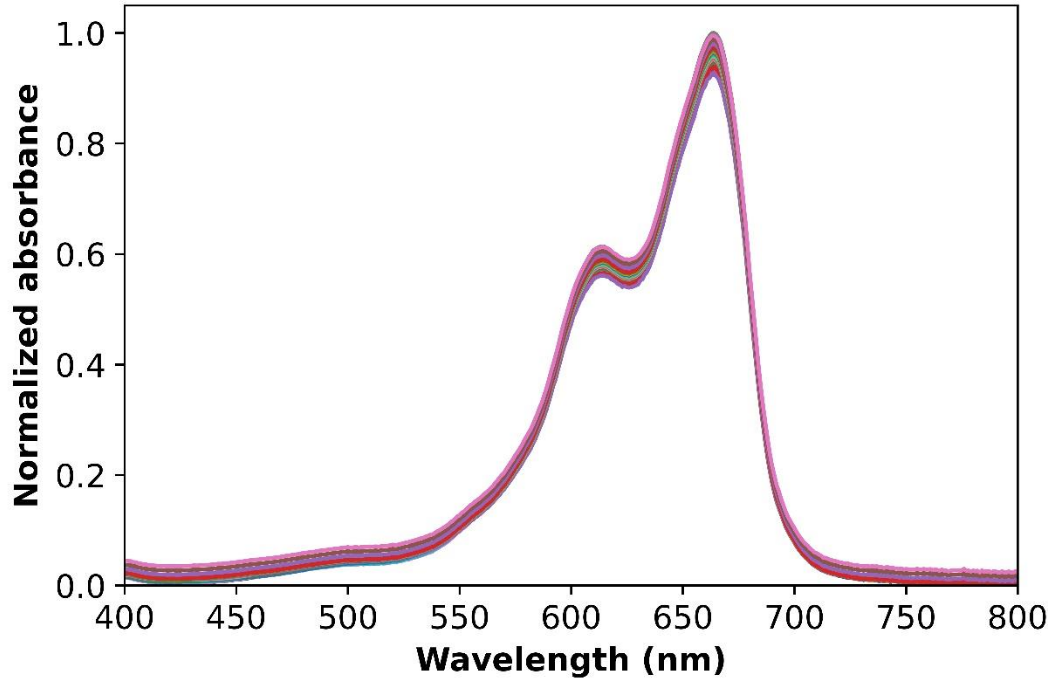

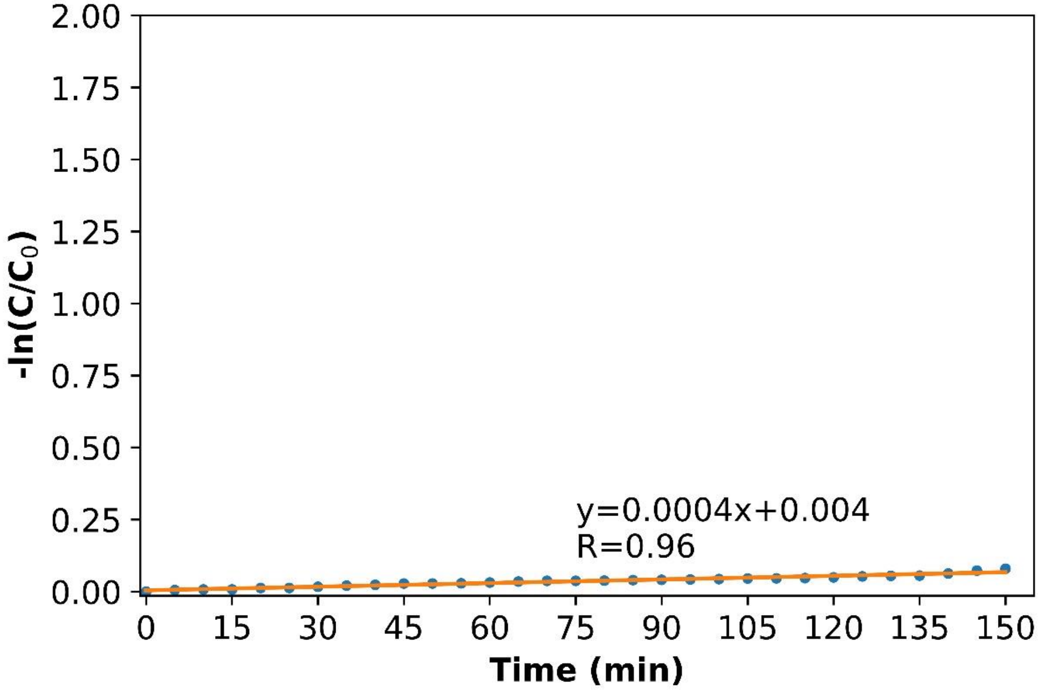

- bCN

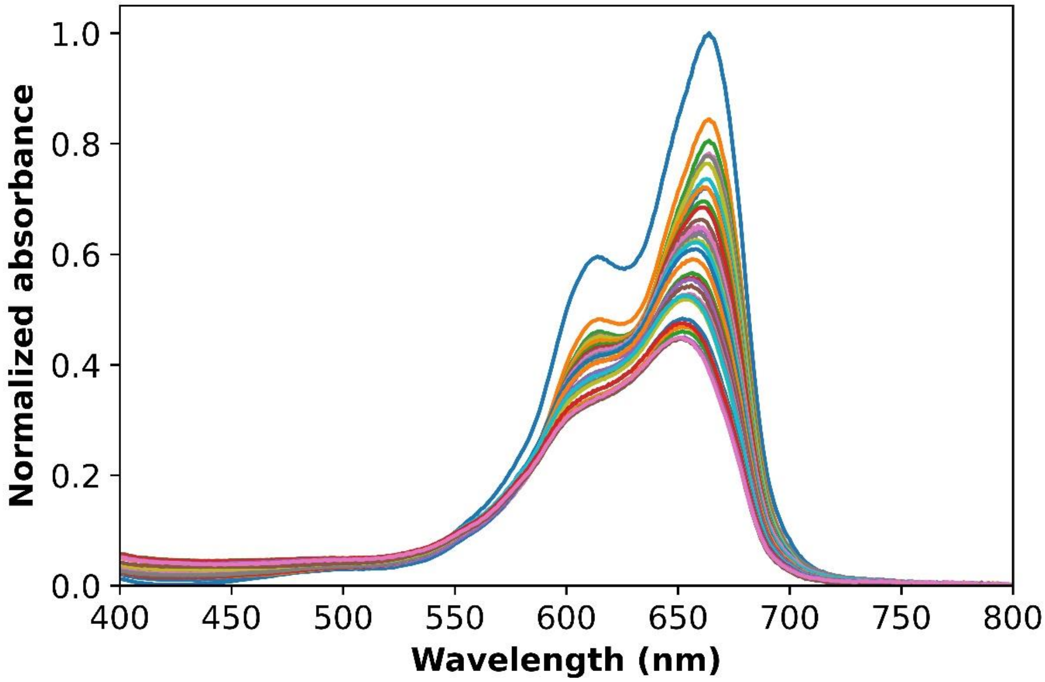
Normalized absorbance
Wavelength (nm)
1.0
0.8
0.6
0.4
0.2
0.0
400
450
500
550
600
650
700
750
800

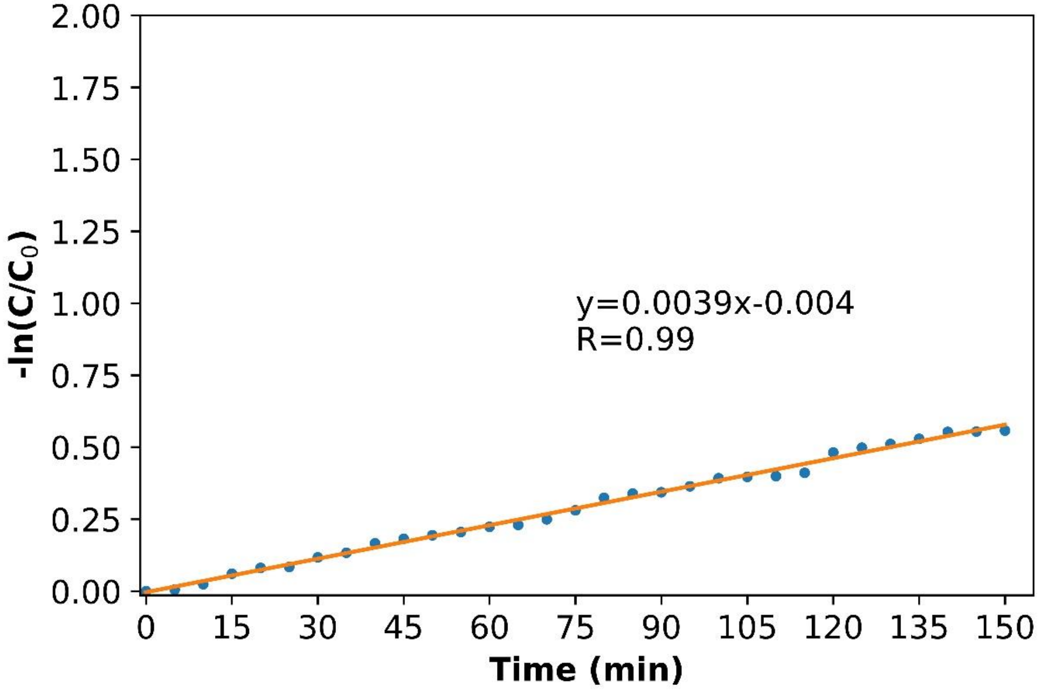
-ln(C/C0)
Time (min)
y=0.0039x-0.004
R=0.99
2.00
1.75
1.50
1.25
1.00
0.75
0.50
0.25
0.00
0
15
30
45
60
75
90
105
120
135
150

- bCN-h

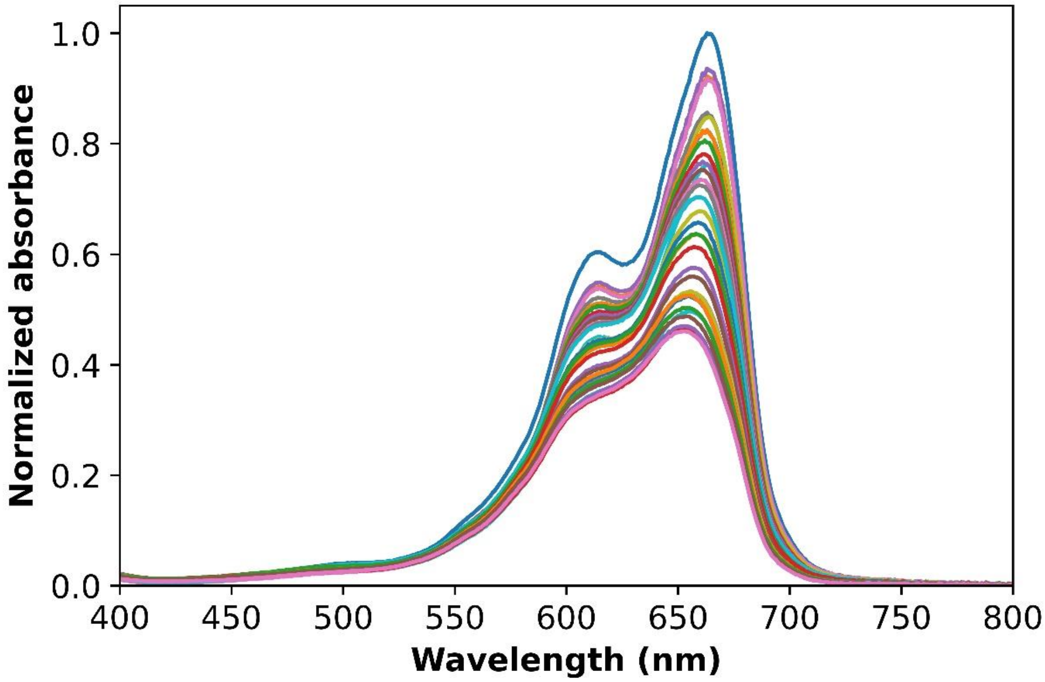
Normalized absorbance
1.0
0.8
0.6
0.4
0.2
0.0
400
450
500
550
600
650
700
750
800
Wavelength (nm)

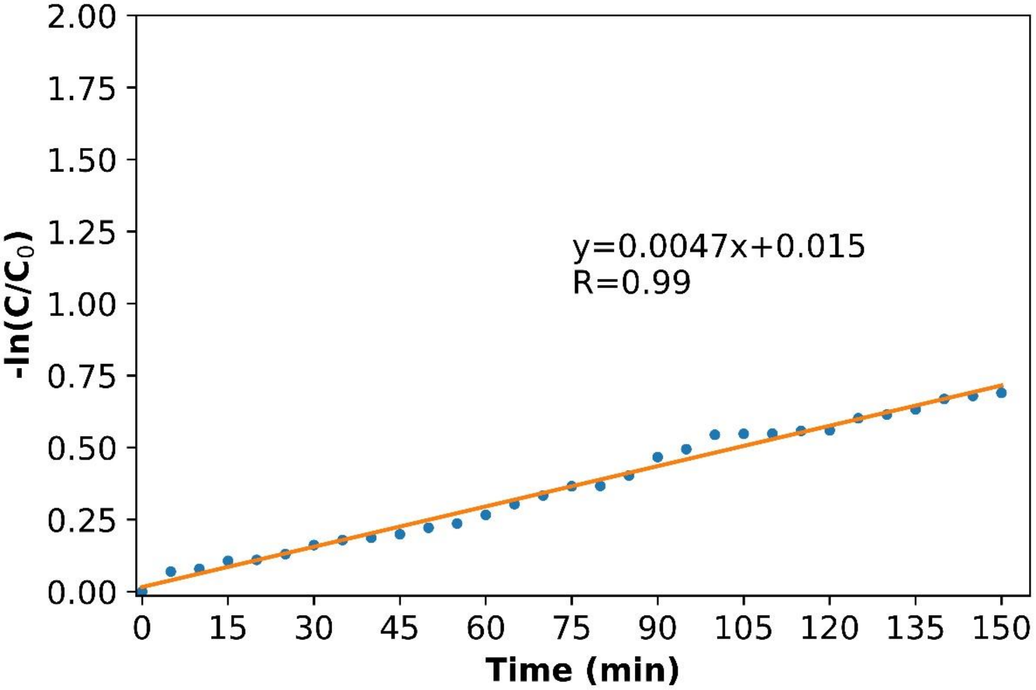
-ln(C/C$_0$)
2.00
1.75
1.50
1.25
1.00
0.75
0.50
0.25
0.00
y=0.0047x+0.015
R=0.99
0
15
30
45
60
75
90
105
120
135
150
Time (min)

- bCN-S

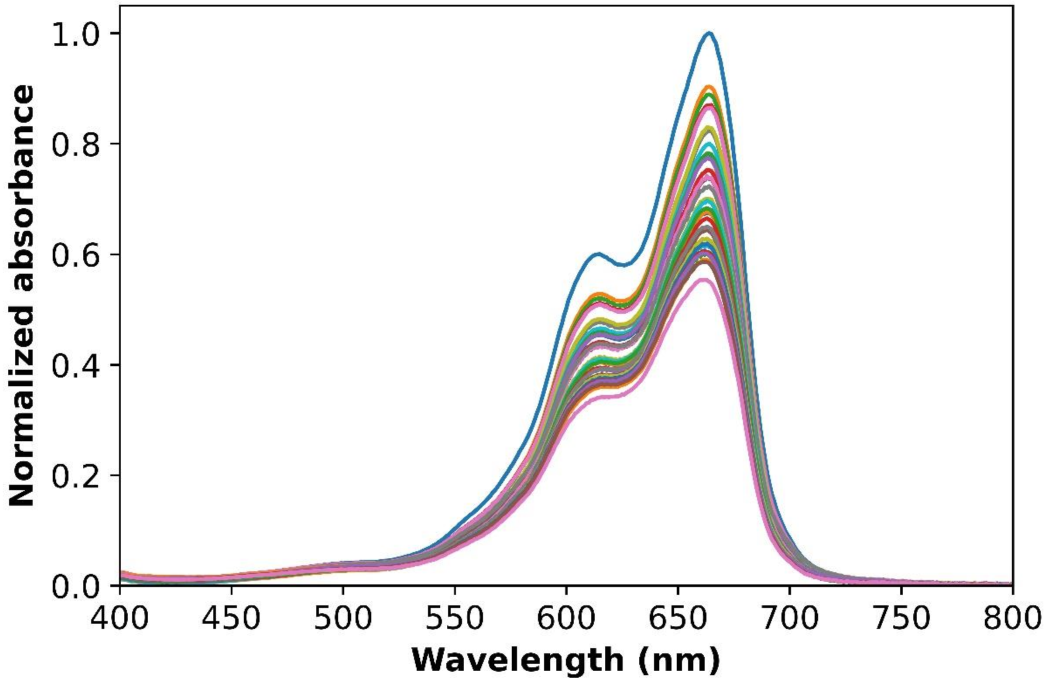

Normalized absorbance
0.0
0.2
0.4
0.6
0.8
1.0
400
450
500
550
600
650
700
750
800
Wavelength (nm)

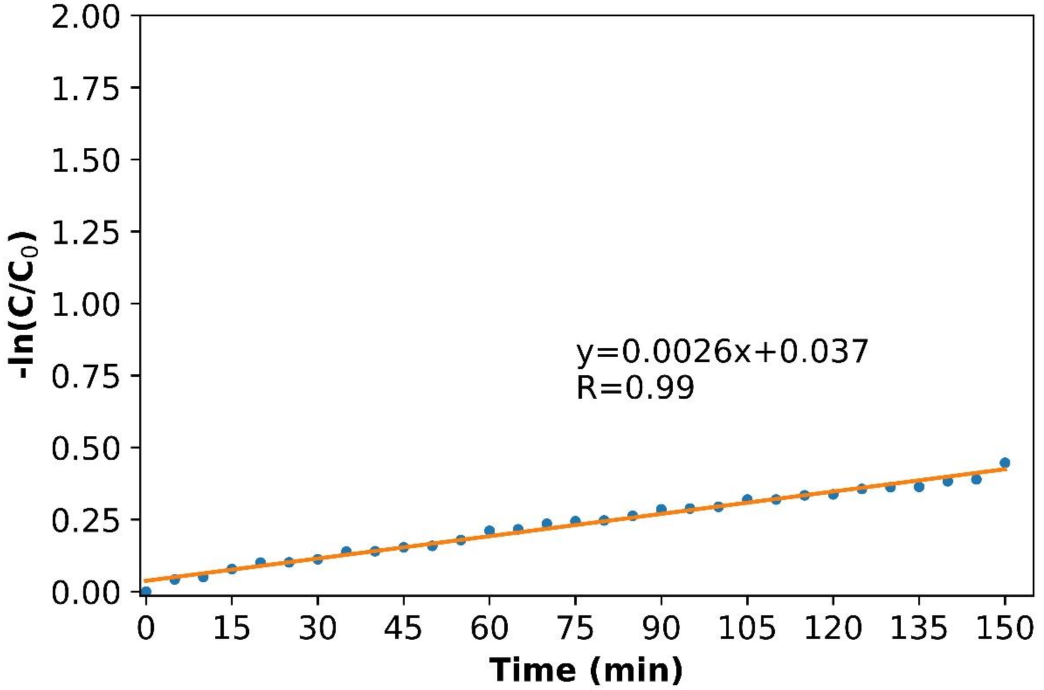

-ln(C/C0)
0.00
0.25
0.50
0.75
1.00
1.25
1.50
1.75
2.00
y=0.0026x+0.037
R=0.99
0
15
30
45
60
75
90
105
120
135
150
Time (min)

- bCN-B

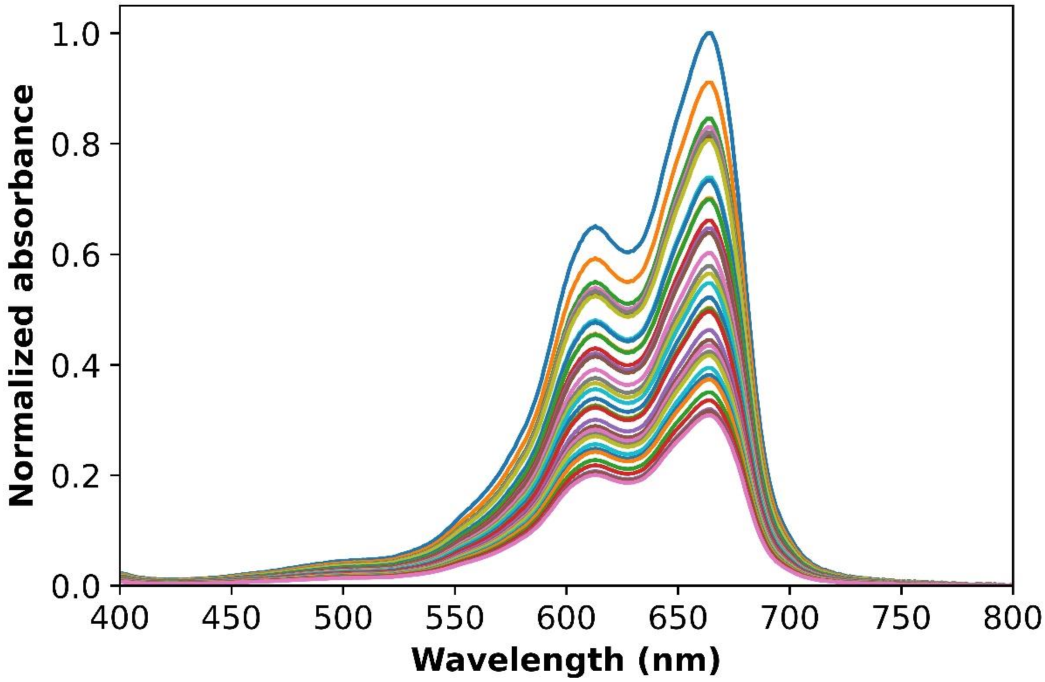

Normalized absorbance
Wavelength (nm)

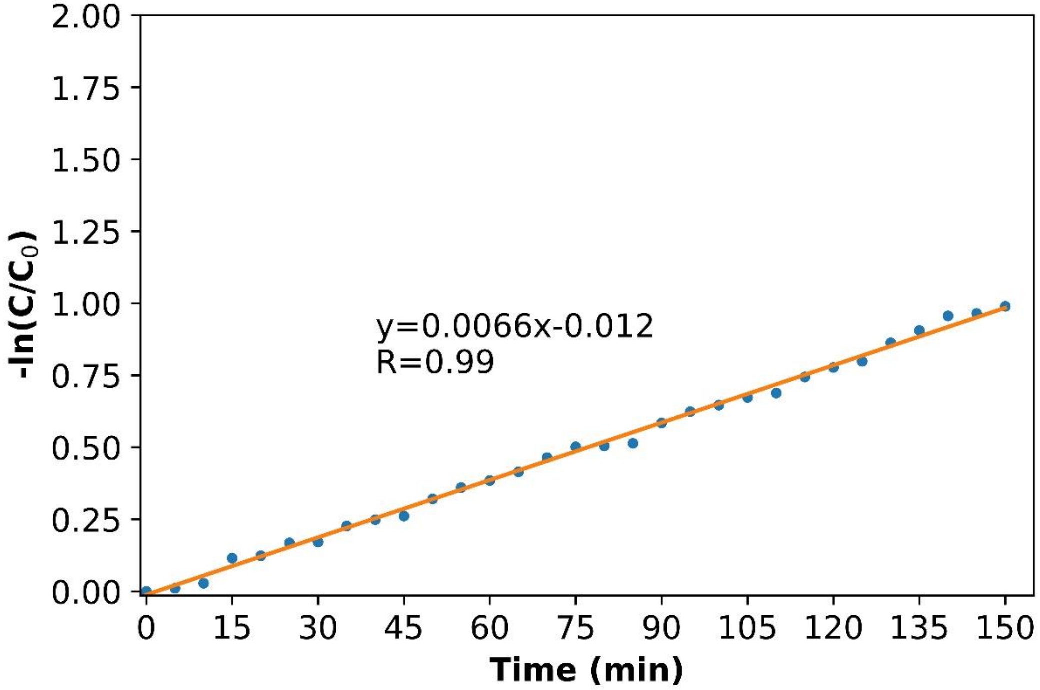

-ln(C/C0)
Time (min)
y=0.0066x-0.012
R=0.99

- eCN-h

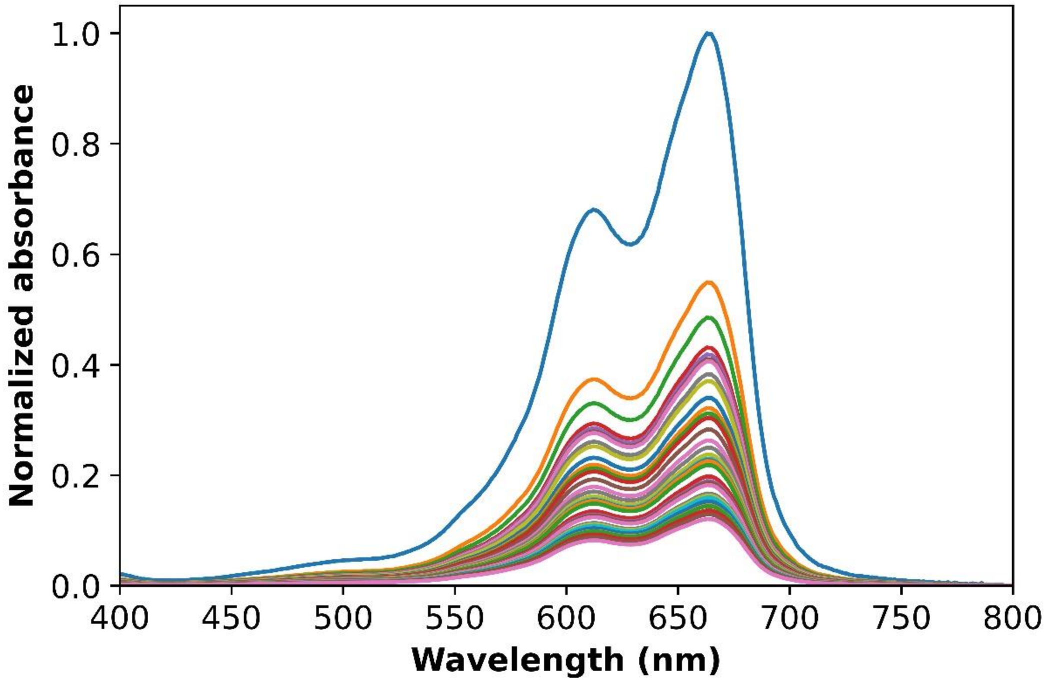
Normalized absorbance
1.0
0.8
0.6
0.4
0.2
0.0
400
450
500
550
600
650
700
750
800
Wavelength (nm)

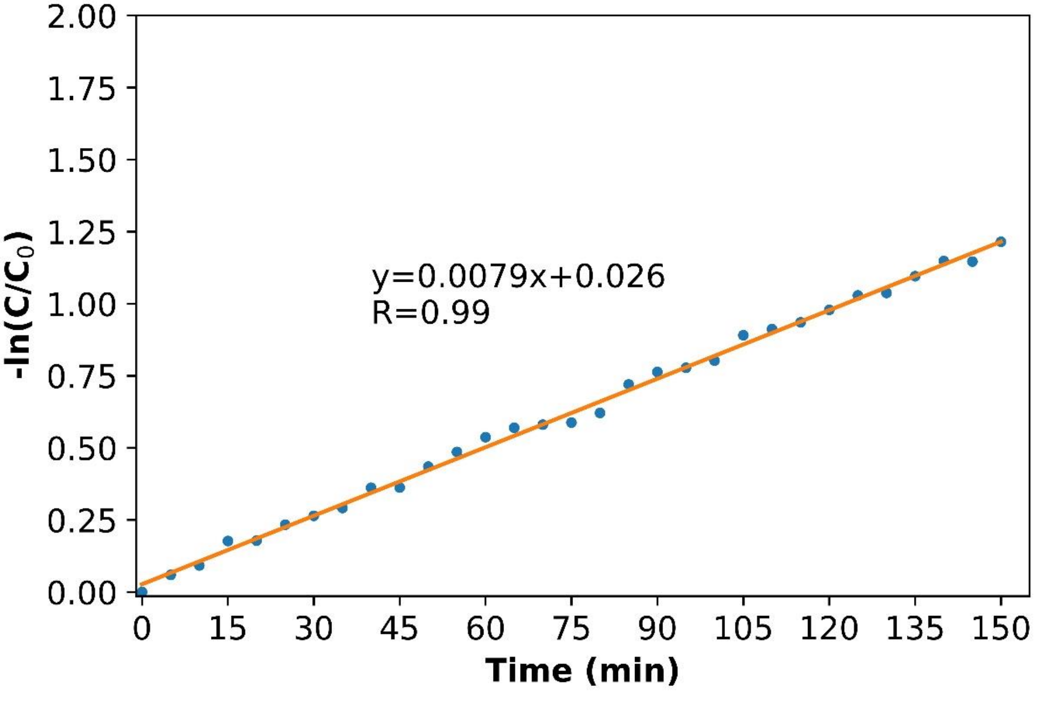
2.00
1.75
1.50
1.25
1.00
0.75
0.50
0.25
0.00
-ln(C/C$_0$)
y=0.0079x+0.026
R=0.99
0
15
30
45
60
75
90
105
120
135
150
Time (min)

- eCN-S

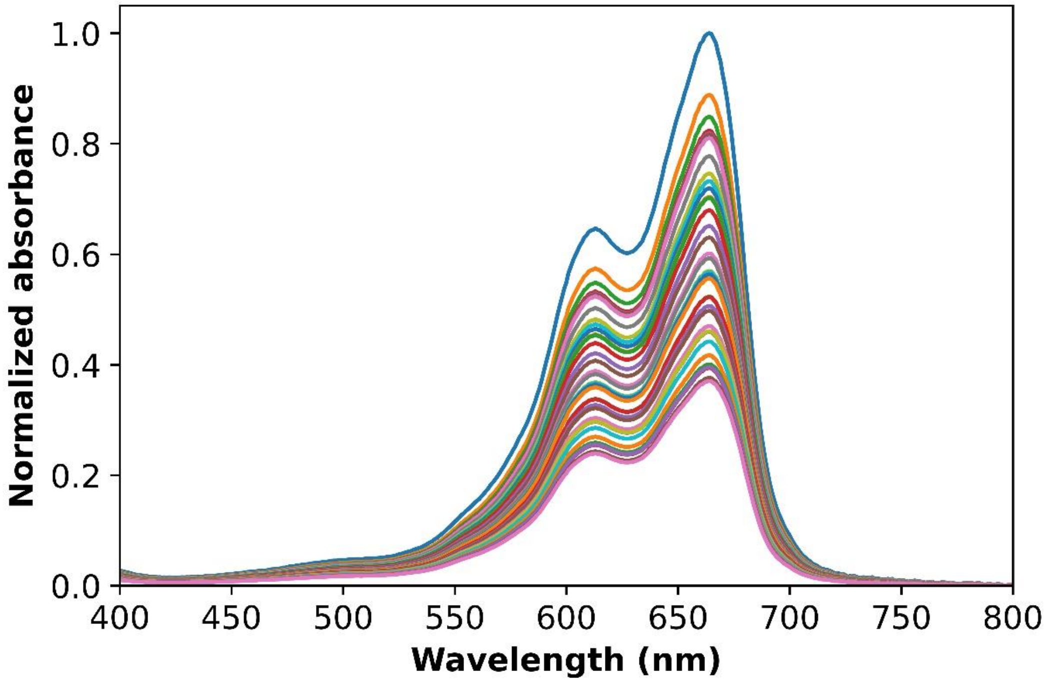

Normalized absorbance
Wavelength (nm)

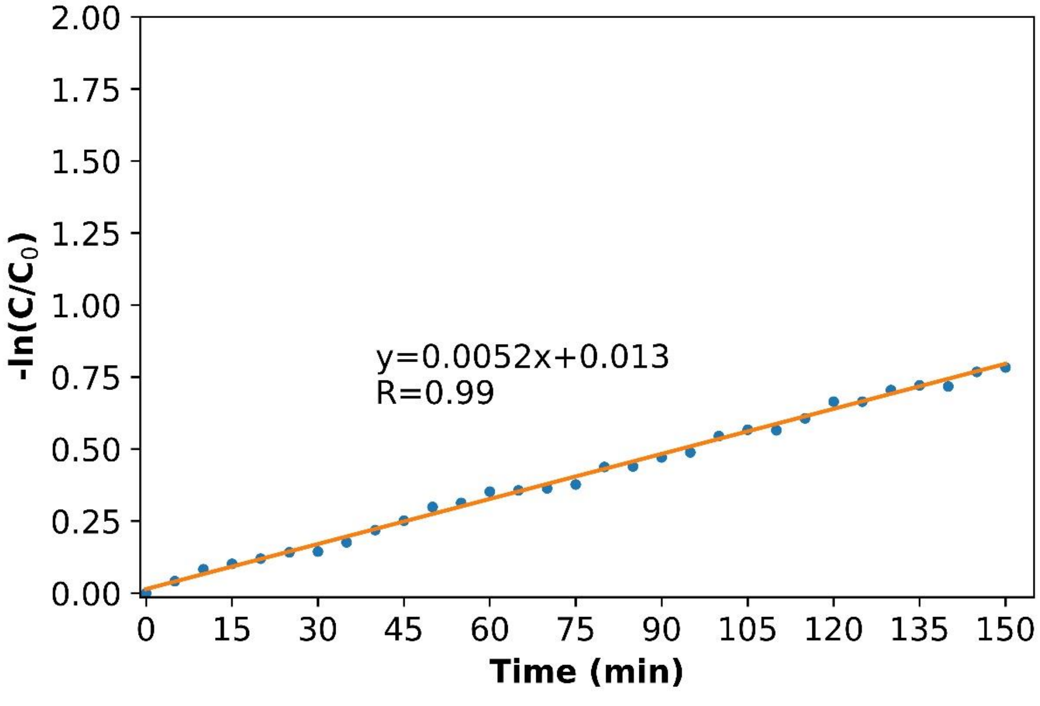

y=0.0052x+0.013
R=0.99
-ln(C/C0)
Time (min)

- CN-B/h-50%

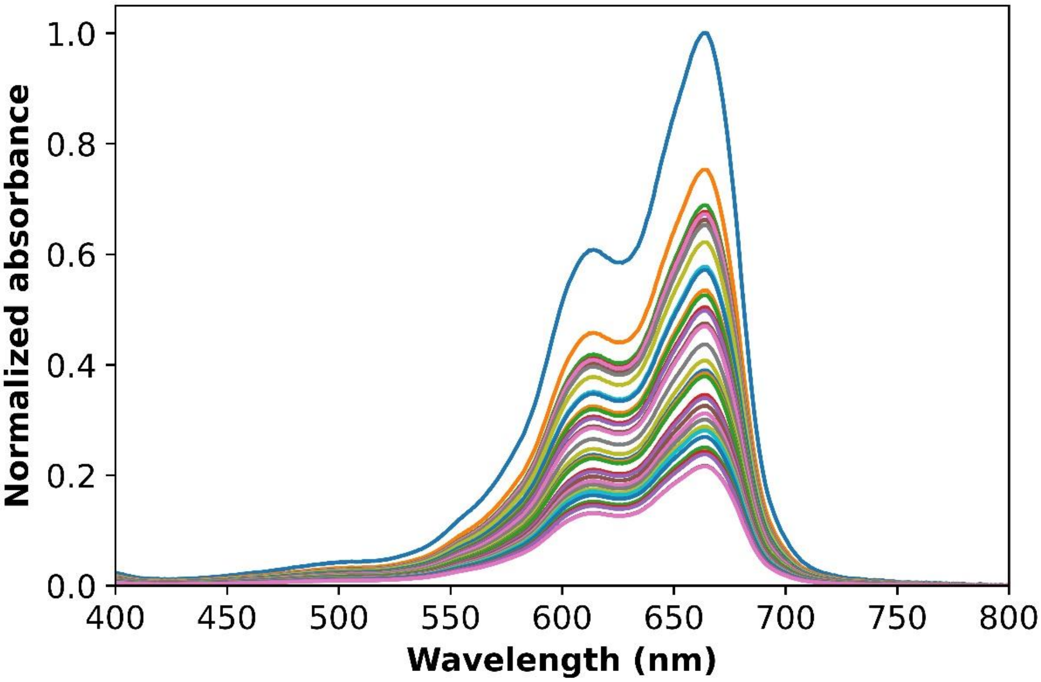
Normalized absorbance
Wavelength (nm)
1.0
0.8
0.6
0.4
0.2
0.0
400
450
500
550
600
650
700
750
800

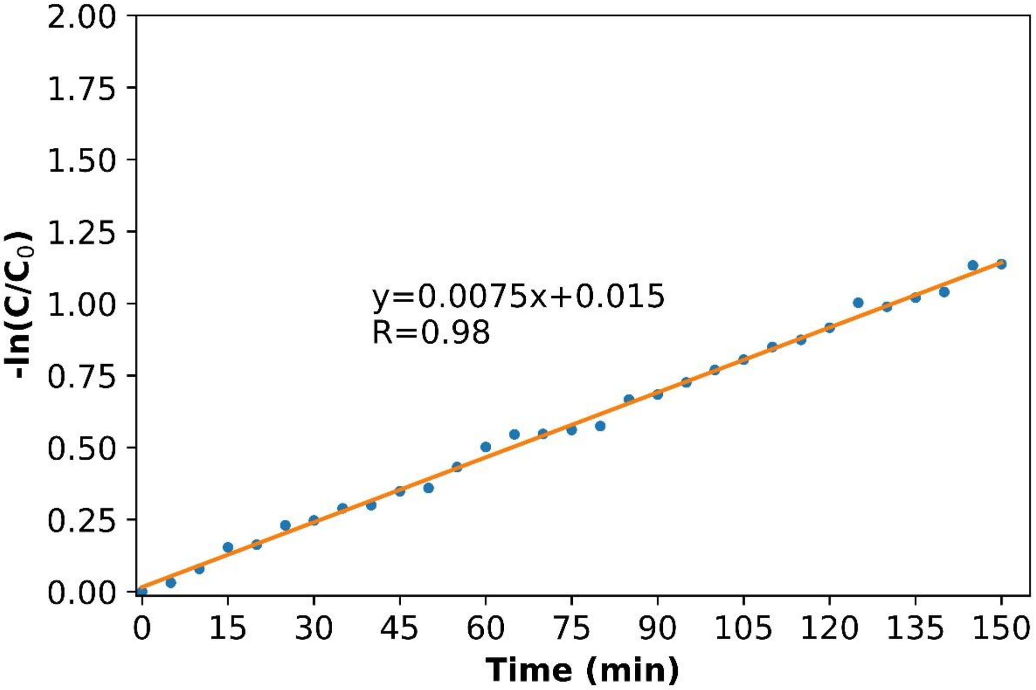
-ln(C/C0)
Time (min)
y=0.0075x+0.015
R=0.98
2.00
1.75
1.50
1.25
1.00
0.75
0.50
0.25
0.00
0
15
30
45
60
75
90
105
120
135
150

- CN-B/S-50%

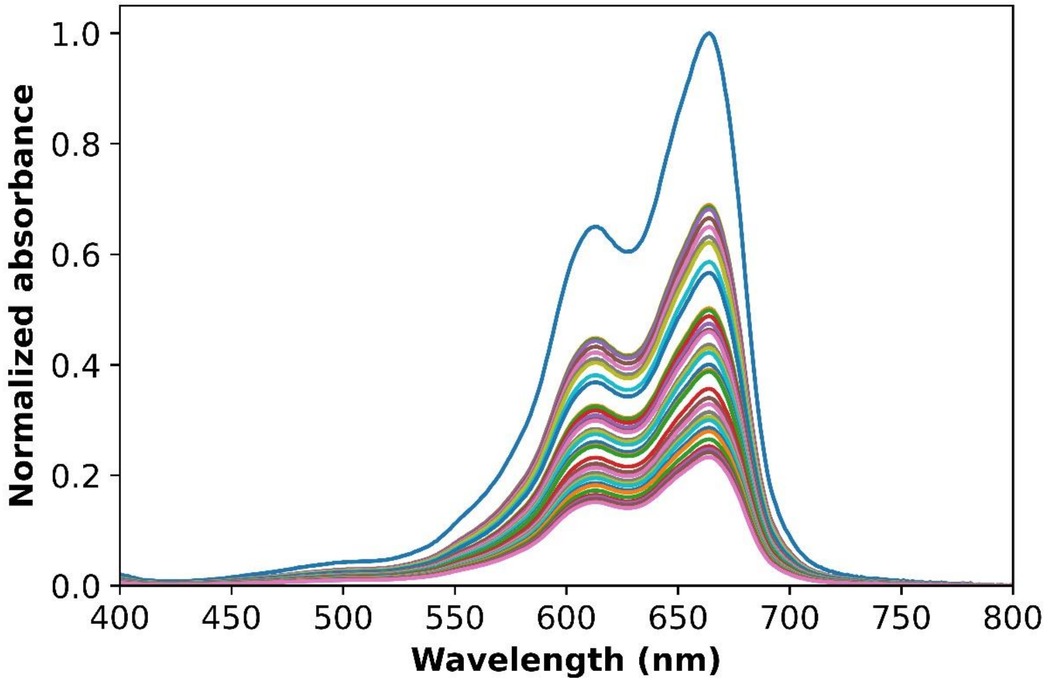
Normalized absorbance
Wavelength (nm)
0.0
0.2
0.4
0.6
0.8
1.0
400
450
500
550
600
650
700
750
800

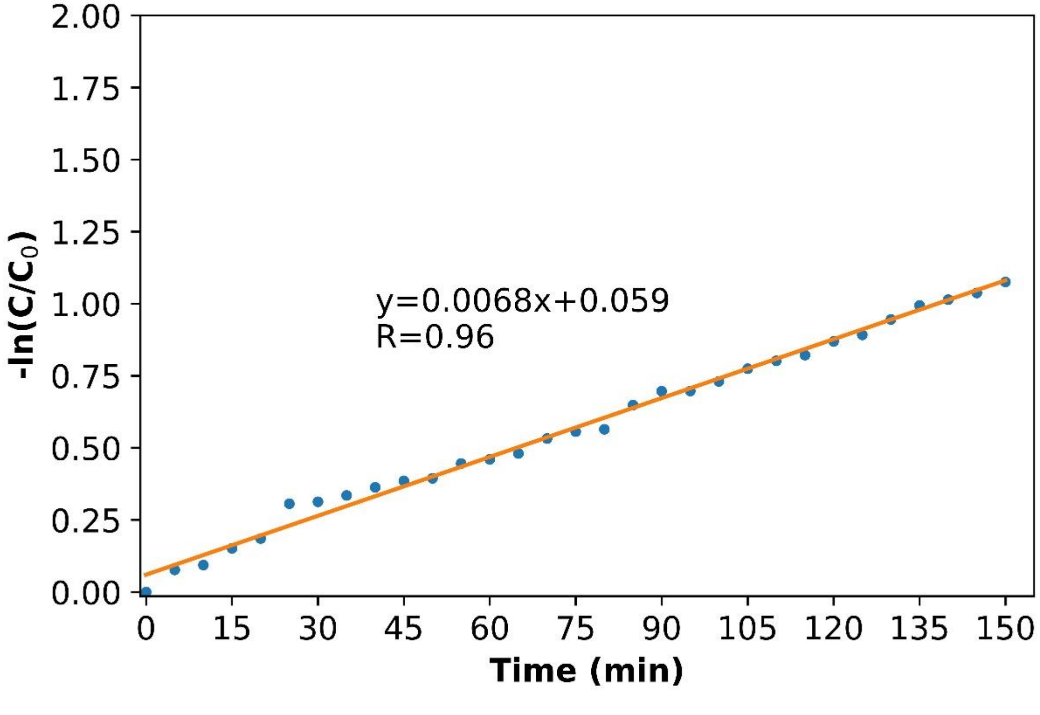
-ln(C/C0)
Time (min)
y=0.0068x+0.059
R=0.96
0.00
0.25
0.50
0.75
1.00
1.25
1.50
1.75
2.00
0
15
30
45
60
75
90
105
120
135
150

- CN-h/S-25%

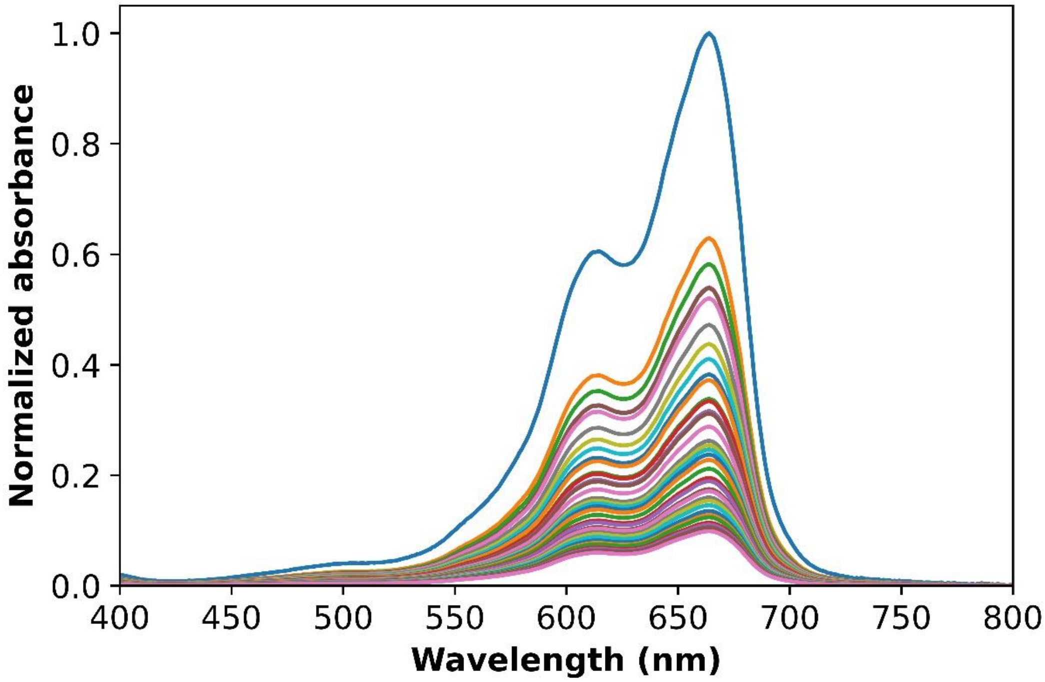

Normalized absorbance
Wavelength (nm)

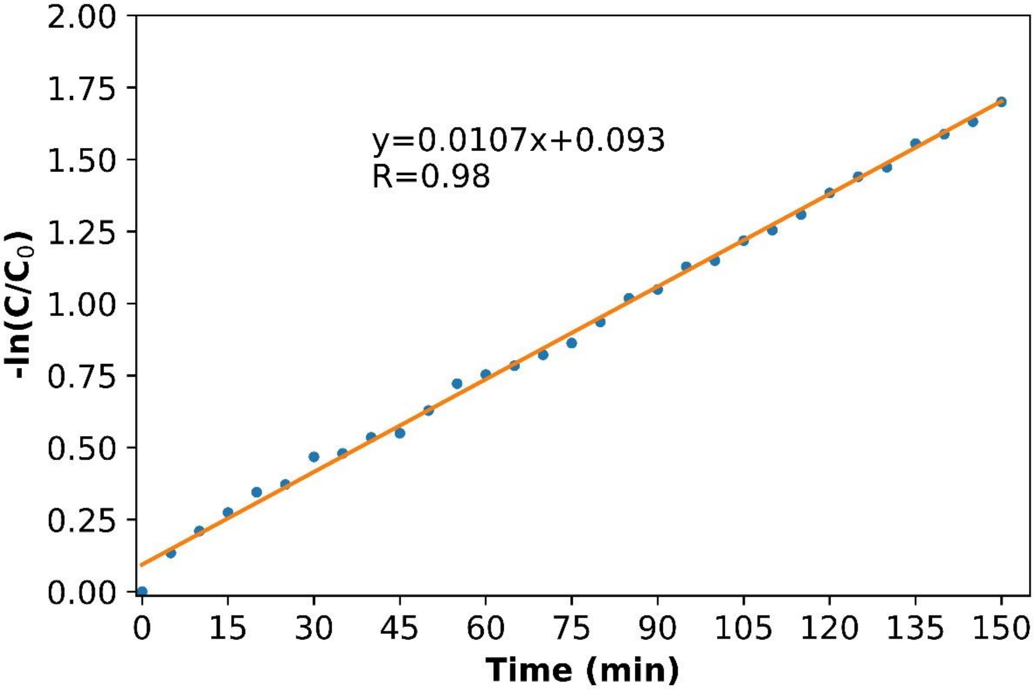

y=0.0107x+0.093
R=0.98
-ln(C/C0)
Time (min)

- CN-h/S-50%

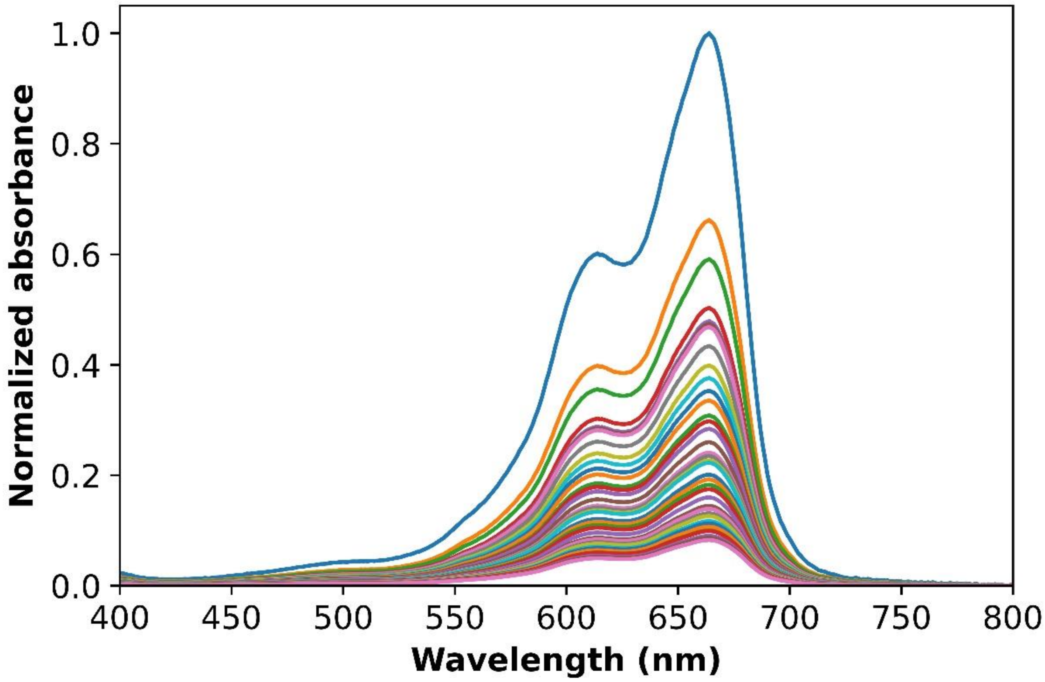
Normalized absorbance
1.0
0.8
0.6
0.4
0.2
0.0
400
450
500
550
600
650
700
750
800
Wavelength (nm)

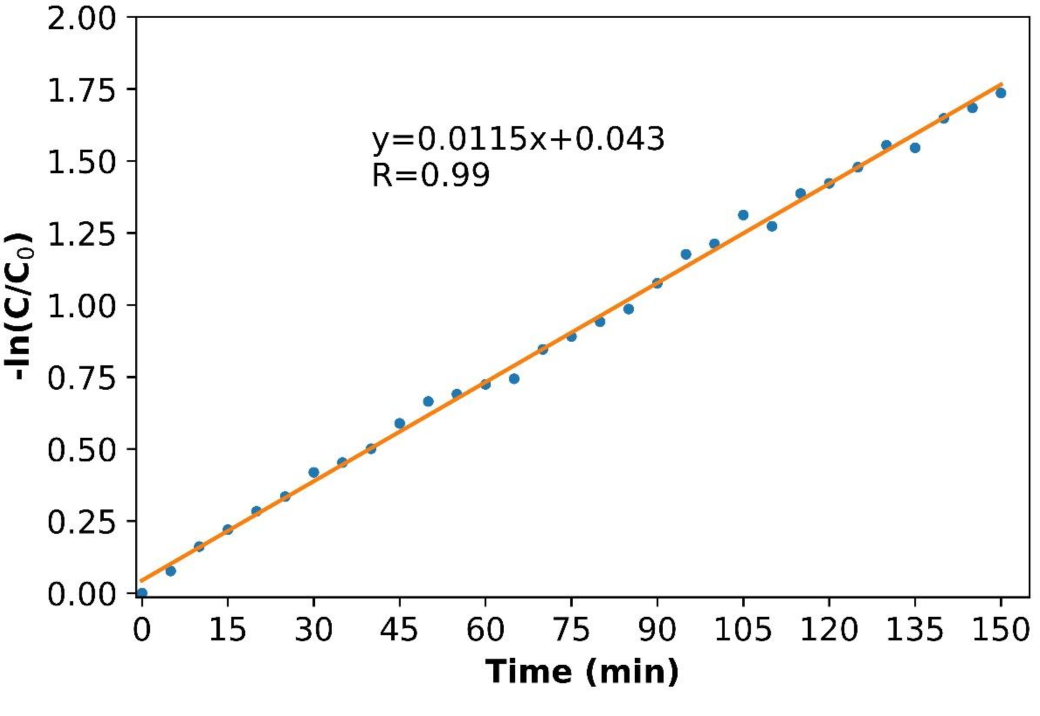
y=0.0115x+0.043
R=0.99
-ln(C/C0)
2.00
1.75
1.50
1.25
1.00
0.75
0.50
0.25
0.00
0
15
30
45
60
75
90
105
120
135
150
Time (min)

- CN-h/S-75%

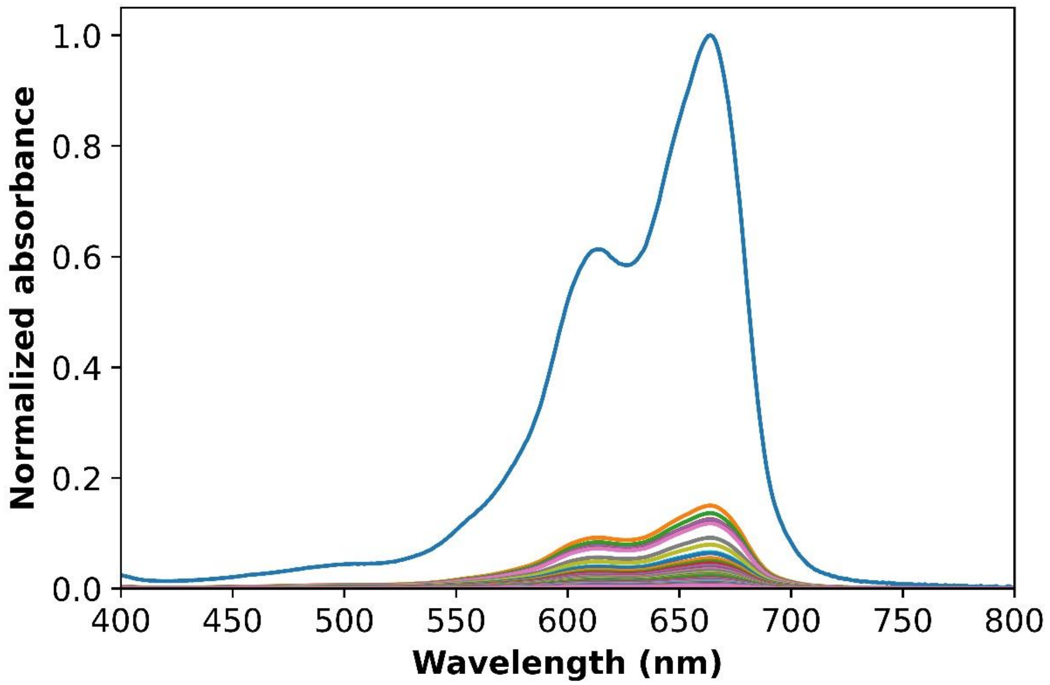
Normalized absorbance
1.0
0.8
0.6
0.4
0.2
0.0
400
450
500
550
600
650
700
750
800
Wavelength (nm)

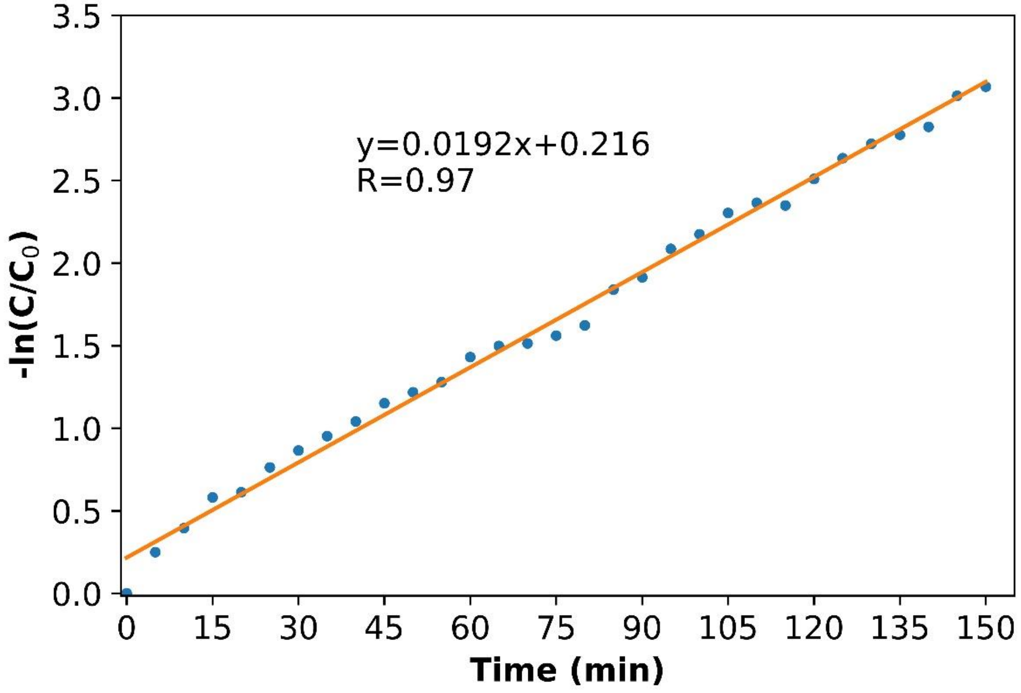
y=0.0192x+0.216
R=0.97
-ln(C/C0)
3.5
3.0
2.5
2.0
1.5
1.0
0.5
0.0
0
15
30
45
60
75
90
105
120
135
150
Time (min)

Valence band position

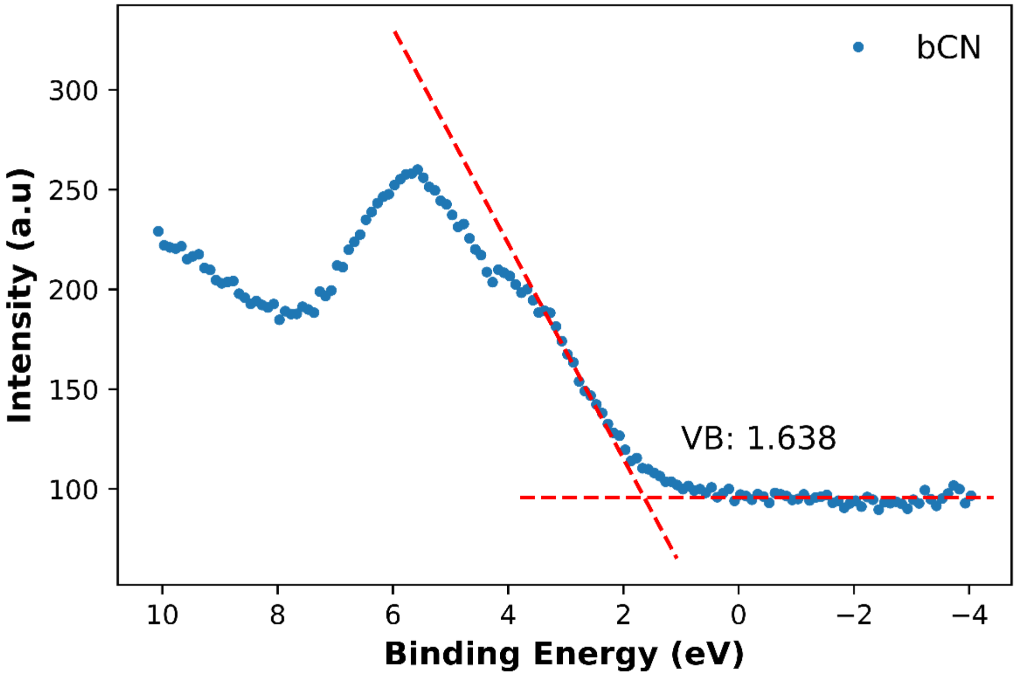

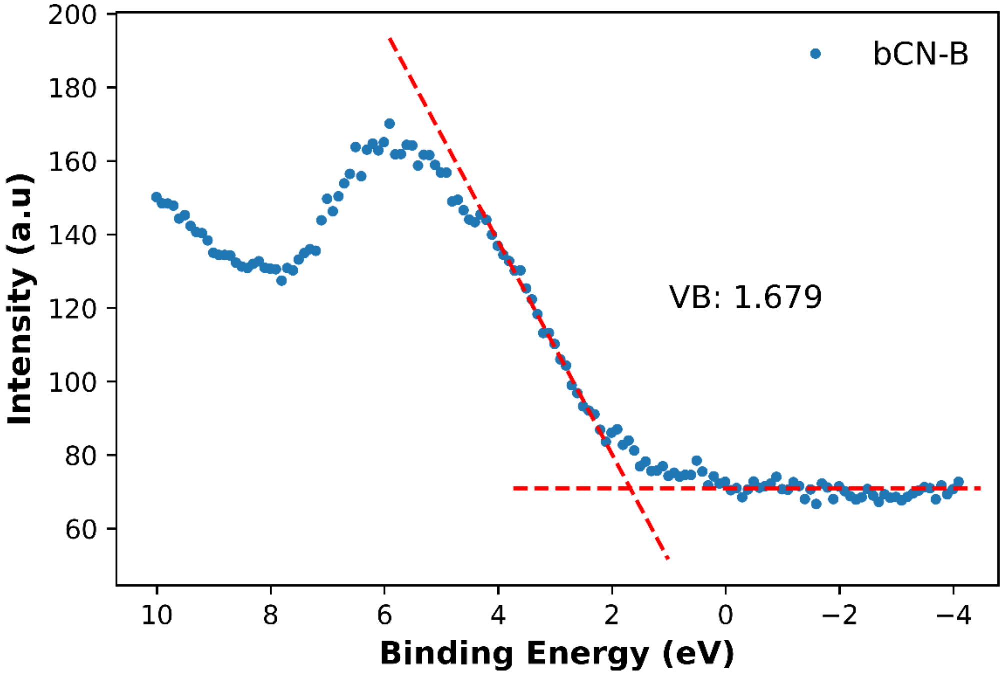

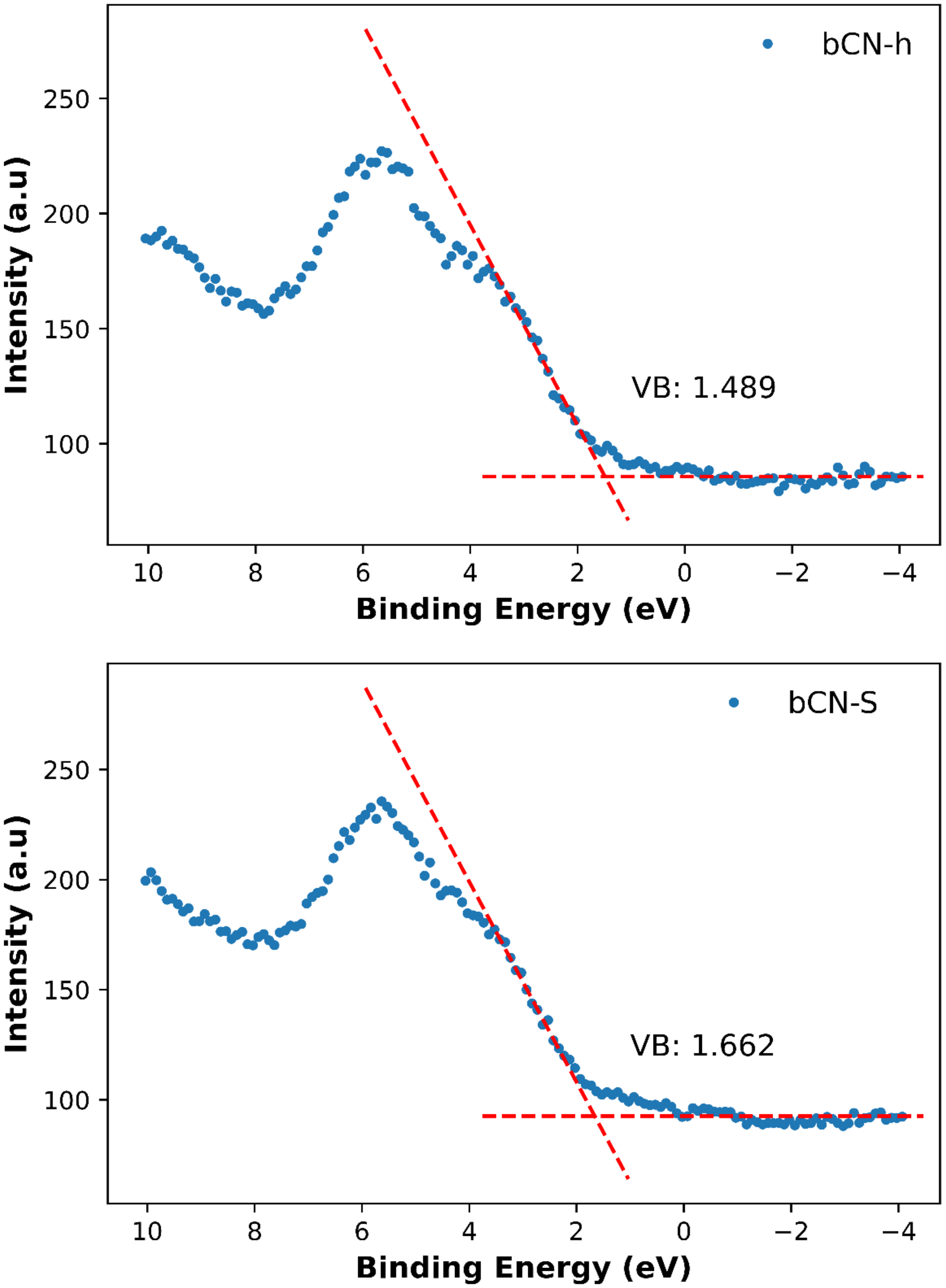
bCN-h
VB: 1.489
Intensity (a.u)
Binding Energy (eV)
bCN-S
VB: 1.662
Intensity (a.u)
Binding Energy (eV)

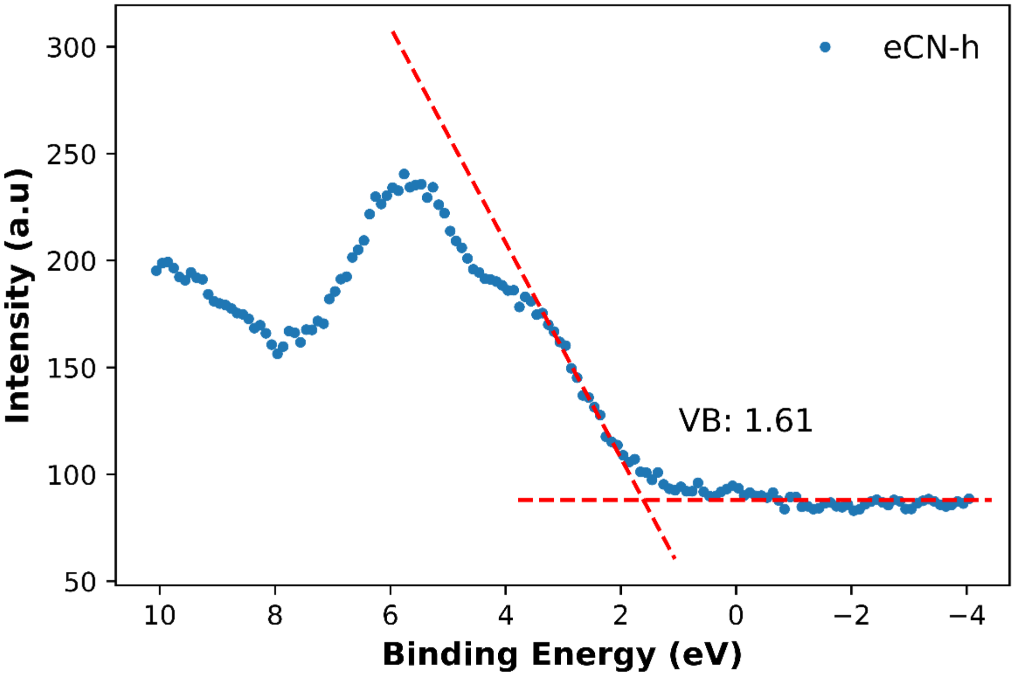
eCN-h
VB: 1.61
Intensity (a.u)
Binding Energy (eV)
300
250
200
150
100
50
10
8
6
4
2
0
−2
−4

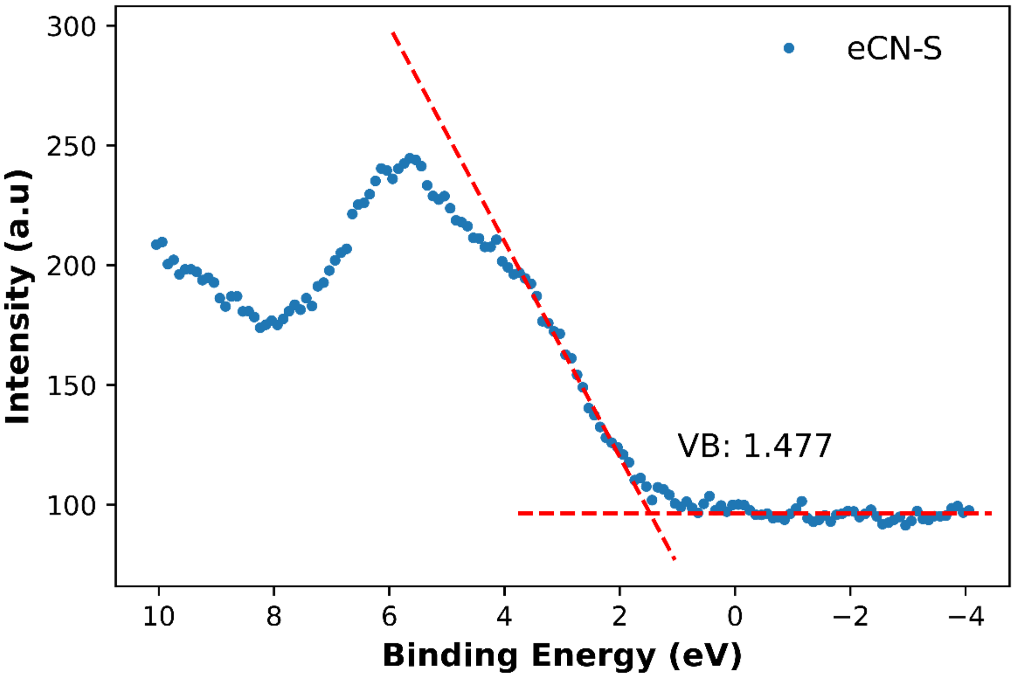
eCN-S
VB: 1.477
Intensity (a.u)
Binding Energy (eV)
300
250
200
150
100
10
8
6
4
2
0
−2
−4

Table S1. Surface atomic composition determined by XPS. No other elements were clearly detectable.

Sample	Composition	atm. %	Ratio C/N
**bCN**	O	7.8	1.53
	C	55.8	
	N	36.4	
**bCN-B**	O	7.9	1.31
	C	51.7	
	N	39.4	
	B	≤1	
**bCN-h**	O	7.2	1.38
	C	53.8	
	N	39.0	
**eCN-h**	O	6.4	1.28
	C	52.6	
	N	41.0	
**bCN-S**	O	8.4	1.49
	C	54.8	
	N	36.8	
**eCN-S**	O	6.5	1.20
	C	50.9	
	N	42.5	
**CN-h/S-75%**	O	3.3	1.01
	C	48.6	
	N	48.1	
